# Immune checkpoint blockade for locally advanced or recurrent/metastatic cervical cancer: An update on clinical data

**DOI:** 10.3389/fonc.2022.1045481

**Published:** 2022-12-22

**Authors:** Zhuo Song, Kun Zou, Lijuan Zou

**Affiliations:** ^1^ Department of Radiation Oncology, The Second Affiliated Hospital, Dalian Medical University, Dalian, Liaoning, China; ^2^ Department of Radiation Oncology, The First Affiliated Hospital, Dalian Medical University, Dalian, Liaoning, China

**Keywords:** cervical cancer, immunotherapy, locally advanced, recurrent metastatic, immune checkpoint, programmed cell death protein 1 (PD-1)

## Abstract

Immunotherapy has shown great promise in the field of oncology, and recent clinical trials have illustrated that immune checkpoint blockade (ICB) is safe and effective at treating a range of tumor types. Cervical cancer (CC) is the fourth most common malignancy in women. However, first-line treatments for locally advanced cervical cancer (LACC) and recurrent/metastatic (R/M) CC have limited efficacy. Thus, it is necessary to explore new treatment approaches. The National Comprehensive Cancer Network (NCCN) currently recommends pembrolizumab, a programmed cell death protein 1 (PD-1) monoclonal antibody, as a first line therapy for individuals with R/M CC. This study reviews the progress of ICB therapy for LACC and R/M CC and describes the current status of the combination of ICB therapy and other therapeutic modalities, including radiotherapy, chemotherapy, targeted therapy, and other immunotherapies. The focus is placed on studies published since 2018 with the aim of highlighting novel CC-specific immunotherapeutic approaches and treatment targets.

## 1 Introduction

Cervical cancer (CC) has the fourth-highest incidence of all common tumors and is the fourth-highest cause of tumor-related mortality in women (1). In 2020, there were >600,000 new cases of CC and 340,000 CC-related fatalities worldwide (1). In China, the incidence of CC has been increasing annually since 2000, with 111,820 new cases and 61,579 deaths expected in 2022, despite the adoption of preventive vaccines and screenings (2, 3). In developing countries where routine screening is not available, more than 70% of CC are already advanced or metastatic at the time of diagnosis (4, 5). Patients diagnosed with late-stage disease have poor survival rates and limited responsiveness to current treatment modalities.

Concurrent chemoradiotherapy (CCRT) is the standard of care for patients with locally advanced cervical cancer (LACC), also known as International Federation of Gynecology and Obstetrics (FIGO) 2018 stage IB3, IIA2-IVA (6). There are no significant differences in the prognosis of squamous and adenocarcinoma patients who are receiving CCRT, and the overall survival (OS) rate is longer than it is for patients receiving radiotherapy alone (7, 8). While CCRT is also effective at managing LACC and the 5-year survival rate among treated patients is 65%, nearly half of patients experience recurrent or metastases (R/M) within 2 years after the initial treatment (9, 10). Once R/M occurs, the 5-year survival rate declines to 17% (11). Thus, there is a need for improved treatment modalities. To address this issue, studies have explored the use of CCRT plus induction, consolidation chemotherapy, or targeted therapy. A phase II study (NCT01973101) found that neoadjuvant chemotherapy with Gemcitabine plus Cisplatin before CCRT was not as effective as CCRT alone (12). A phase III clinical study (OUTBACK Trial) showed that four cycles of adjuvant chemotherapy with Paclitaxel plus Carboplatin following standard CCRT did not prolong patient survival (13). Most current studies do not recommend adjuvant chemotherapy for LACC patients (14). Targeted anti-angiogenesis inhibitor therapy with Endostar in combination with CCRT increased the distance metastasis-free survival (DMFS) but did not improve progression-free survival (PFS) (15). The RTOG 0417 trial showed positive outcomes with 3-year OS and DFS rates of 81.3% and 68.7%, respectively, for Bevacizumab in combination with CCRT (16). However, these treatments have limited efficacy and are associated with some negative results.

R/M CC patients are typically prescribed systemic therapy and the 5-year survival rate is only 17%. Patients with squamous and adenocarcinoma or adenosquamous carcinoma have similar survival rates following chemotherapy (11, 17). The GOG-204 trial showed that treatment with Paclitaxel and the Cisplatin/Topotecan regimen combined with Bevacizumab resulted in a median OS of 16.8 months and a median post-progression OS of approximately 8.4 months (18). This was previously the first-line treatment option for R/M CC and extended OS by 3.5 months compared to chemotherapy alone. However, first-line treatments have limited efficacy and restricted second-line treatment options are currently available, demonstrating a need for new treatment modalities. There have been several breakthroughs in immunotherapy and this has become a powerful new mechanism for treatment after surgery and radiotherapy, especially among patients with advanced, recurrent, refractory, or metastatic tumors. Several immune checkpoint drugs specific to CC are currently in clinical trials, some of which have good efficacy and safety. For example, the KEYNOTE 826 trial showed that Pembrolizumab plus chemotherapy with or without Bevacizumab further improved OS by 7.6 months (19). This regimen was the first-line treatment option recommended by the 2022 National Comprehensive Cancer Network (NCCN) guidelines.

This review summarizes the current status of immune checkpoint blockades (ICBs) studied in LACC and R/M CC over the past 5 years, including monotherapy and combination therapy. The aim of this study was to find appropriate first-, second-, and later-line treatment options and identify patient populations who could potentially benefit from immunotherapy.

## 2 Immunological mechanism of CC

### 2.1 Tumorigenesis and establishment of the CC microenvironment

CC is a human papillomavirus (HPV)-associated neoplasm. The high-risk subtypes (hrHPVs), HPV types 16 and 18, are responsible for >70% of invasive CC (20). While 85–90% of hrHPV infections clear spontaneously, 10–15% persist (21), suggesting that the development of CC requires other cofactors associated with HPV-infected cells to guide the development of the tumor microenvironment (TME) and cause viral persistence, multiplication and tumor progression (21). Using transcriptomics, single-cell analysis, and other high-throughput sequencing methods, studies have confirmed the importance of T cells in tumor immunotherapy (22). For example, some tumor cells are shown to express programmed cell death ligand-1 (PD-L1), which binds to the programmed cell death protein 1 (PD-1) receptor on the surface of T cells and inhibits their activity (23). Meanwhile, expression of the HPV E6 and E7 oncogenes upregulates PD-L1 by suppressing p53 and Rb (24, 25). The immunosuppressive microenvironment caused by HPV infection is a potential target for immunotherapy.

### 2.2 The rationale and study status of ICB

**Figure 1:**
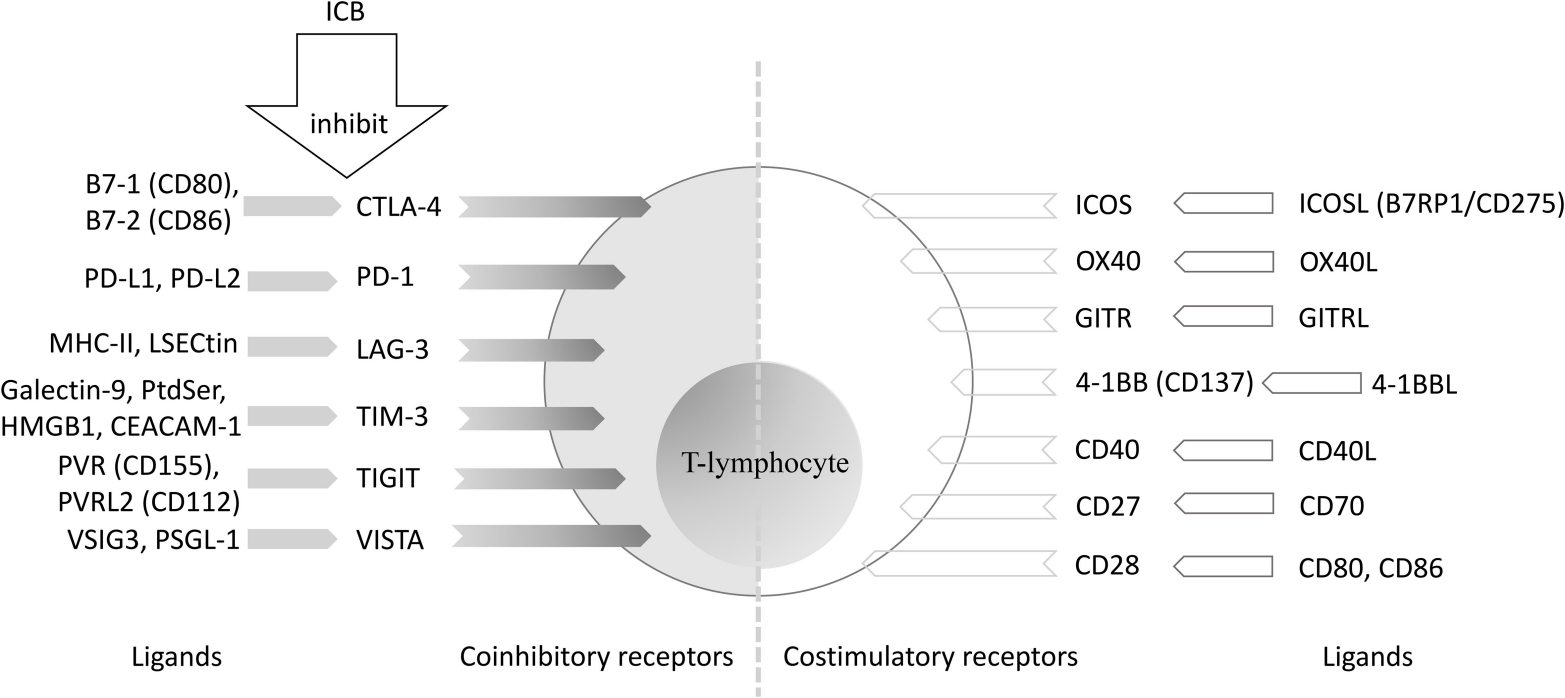
T lymphocyte-associated co-stimulatory and co-inhibitory molecules. ICB, immune checkpoint blockade; CTLA-4, cytotoxic T-lymphocyte-associated protein 4; PD-1, programmed cell death protein 1; PD-L1, programmed cell death ligand-1; PD-L2, programmed cell death ligand-2; MHC: major histocompatibility complex; LSECtin, liver and lymph node sinusoidal endothelial cell C-type lectin; LAG-3, Lymphocyte Activation Gene-3; PtdSer, phosphatidylserine; HMGB1, High Mobility Group Protein 1; CEACAM-1, carcinoembryonic antigen-related cell adhesion molecule-1; TIM-3, T cell immunoglobulin and mucin-containing molecule 3; PVR: poliovirus receptor; PVRL2, poliovirus receptor-related protein 2; TIGIT, T cell immunoglobulin and ITIM domains; VISTA, V-domain immunoglobulin suppressor of T cell activation; PSGL-1, P-selectin glycoprotein ligand-1; VSIG3, V-set and immunoglobulin domain-containing 3; ICOS, inducible T-cell co-stimulator; B7RP1, B7-related protein 1; GITR, glucocorticoid-induced tumor necrosis factor receptor; 4-1BB, tumor necrosis factor receptor superfamily member 9.

Current ICB activates tumor-reactive T cells by downregulating the inhibitory signaling pathway or overcoming regulatory mechanisms that prevent T cell activation (26). Promising immune targets of ICB that focus on PD-1 and cytotoxic T-lymphocyte-associated protein 4 (CTLA-4), are summarized in **Figure 1** (26–29). These immune checkpoints are primarily located on the surface of T cells, natural killer (NK) cells, dendritic cells (DCs), B cells, monocytes, macrophages, and neutrophils (30). While the biological mechanisms are not fully understood, these checkpoints may still aid in the design of novel ICB drugs and help to identify methods of drug resistance.

The most studied ICB targets include PD-1 (CD279) and its ligand PD-L1 (CD274/B7 homolog 1, B7-H1), CTLA-4 (CD152), and indoleamine 2, 3-dioxygenase 1 (IDO1) (31). Specific ICBs, including Nivolumab, Pembrolizumab, and Atezolizumab, have been designed for some solid tumors including lung cancer and melanoma. It is expected that the drug indications will expand in include the treatment of CC (32–35). Phase III clinical trials related to immune checkpoint drugs specific for LACC and R/M CC are shown in **Table 1**, and Phase I/II clinical trials are illustrated in **Supplementary Tables 1** and **2**, respectively.

**Table 1:** Phase III clinical trial of immune checkpoint drugs for locally advanced or recurrent/metastatic cervical cancer (derived from clinicaltrials.gov, accessed 12 Sep 2022).

NCT Number	Other IDs	Study Populations	Interventions	Targets	Enrollment	Primary Endpoints	Estimated Completion Date
NCT04943627	C-700-03	Recurrent or Metastatic Cervical Cancer	Balstilimab (AGEN2034)	PD-1	6	OS	2021/10/22
NCT04864782	QL1604-301	Stage IVB, Recurrent, or Metastatic Cervical Cancer	QL1604 + Paclitaxel + Cisplatin/Carboplatin	PD-1	458	ORR + PFS	2022/7/1
NCT03635567	MK-3475-826/KEYNOTE-826	Persistent, Recurrent, or Metastatic Cervical Cancer	Pembrolizumab (MK-3475) + Paclitaxel + Cisplatin/Carboplatin ± Bevacizumab	PD-1	600	PFS + OS	2022/11/23
NCT04906993	SHR-1210-III-329	Recurrent/Metastatic Cervical Cancer	Camrelizumab (SHR-1210) + Famitinib Malate	PD-1 + VEGFR-2	440	PFS + OS	2023/5/31
NCT03830866	D9100C00001	FIGO (2009) Stages IB2 to IIB node positive or IIIA-IVA any node	Durvalumab + Cisplatin/Carboplatin + EBRT + Brachytherapy	PD-L1	770	PFS	2023/6/30
NCT05446883	QL1706-301	Recurrent or Metastatic Cervical Cancer	QL1706 + Paclitaxel + Cisplatin/Carboplatin ± Bevacizumab	PD-1 and CTLA-4	498	PFS + OS	2024/6/1
NCT03912415	BCD-100-5	Progressing thru/Recurrent, or Primary Metastatic Cervical Cancer FIGO Stage IVB	BCD-100+ Paclitaxel + Carboplatin/Cisplatin ± Bevacizumab	PD-1	316	OS	2024/12/1
NCT04221945	MK-3475-A18/KEYNOTE-A18/ENGOT-cx11/GOG-3047	FIGO (2014) Stage IB2-IIB (with node-positive disease) or Stages III-IVA	Pembrolizumab + Cisplatin + EBRT + Brachytherapy	PD-1	980	PFS + OS	2024/12/7
NCT04982237		Persistent, Recurrent, or Metastatic Cervical Cancer	AK104 (Cadonilimab) + Paclitaxel + Carboplatin/Cisplatin ± Bevacizumab	PD-1 and CTLA-4	440	PFS + OS	2025/12/30
NCT05173272	KY-2021-109	FIGO (2018) Stage Ib3-IIIc2	Serplulimab (Neoadjuvant Therapy)	PD-1	286	PFS	2028/9/28
NCT05235516	AK104- 305	FIGO (2018) Stage 3A-4A	AK104 (Cadonilimab) + EBRT + Brachytherapy + Cisplatin	PD-1 and CTLA-4	636	PFS	2029/5/1

FIGO, International Federation of Gynecology and Obstetrics; EBRT, external beam radiotherapy; PD-1, programmed cell death protein 1; PD-L1, programmed cell death ligand-1; CTLA-4, cytotoxic T-lymphocyte-associated protein 4; VEGFR-2, vascular endothelial growth factor receptor 2; PFS, progression-free survival; OS, overall survival; ORR, objective response rate.

## 3 ICB monotherapy for R/M CC

The common ICB monotherapy used to treat R/M CC includes anti-PD-1, anti-CTLA-4, anti-PD-L1, and bispecific antibodies. Clinical trials (NCT02315066, NCT04693234) are ongoing for new targets such as the OX40 agonist, Ivolizumab (PF-04518600), anti-T cell immunoglobulin, and the ITIM domains (TIGIT) antibody, Ociperlimab (BGB-A1217) (36, 37). Meanwhile, clinical studies for new drugs, such as NCT05171790 for QL1706 (a PD-1/CTLA-4 bi-specific antibody) are ongoing (38). On June 12, 2018, Pembrolizumab was approved by the U.S. Food and Drug Administration (FDA) for R/M CC with Combined Positive Score (CPS) ≥1 progression on or after chemotherapy, and on June 29, 2022, Cadonilimab was approved by the China National Medical Products Administration (NMPA) for the second-line treatment of R/M CC. Additional drugs are still in clinical trial phases. Furthermore, data on first-line treatments remains lacking. A comparison of published data on immune checkpoint drug monotherapies for R/M CC is shown in **Table 2** (32–35, 38–47).

**Table 2:** Published data on immune checkpoint drug monotherapy for recurrent/metastatic cervical cancer.

Ref.	Trial	Phase	N	Histology	Patient population	Treatment regimens	Primary endpoint
(33)	KEYNOTE-158 NCT02628067 (Multi-location)	II	98	SCC = 93.9% AC = 5.1% ASC = 1.0%	Advanced CC; progression during or intolerance to ≥1 lines of standard therapy	Pembrolizumab 200 mg IV q3w for up to 2 years	ORR, 12.2% (PD-L1+, 14.6%; PD-L1-, 0%)
(39)	KGOG1041 (Korean)	Retrospective	117	SCC = 75.2% AC = 16.2% ASC = 3.4% NEC = 3.4% GCC = 0.9% BSCC = 0.9%	CC; tumor progression during or after the use of ≥1 lines of chemotherapy	Pembrolizumab 200 mg IV q3w until disease progression, unacceptable toxicity, or patient withdrawal occurred	ORR, 9.4% (ECOG ≤1, 18.9%); Safety: AEs, 55 (47.0%) pts; AEs ≥ grade 3, 8 (6.8%) pts; suspicious treatment-related deaths, 2 of 8
(40)	EMPOWER- Cervical 1/GOG-3016/ENGOT-cx9 NCT03257267 (Multi-location)	III	Cemiplimab = 304; Chemotherapy = 304	SCC = 77.8% AC/ASC = 22.2%	R/M CC; disease progression after first- line platinum-containing chemotherapy	Cemiplimab 350 mg IV q3w until PD, unacceptable toxicity, or until 96 weeks; The investigator’s choice of single-agent chemotherapy.	Median OS, 12.0 months vs. 8.5 months;
(41)	NCT03104699 (Multi-location)	II	161	SCC = 62.7% AC = 32.3% ASC = 4.3% Other = 0.6%	R/M CC; disease progression after first- line platinum-based treatment regimen	Balstilimab 3mg/kg IV q2w for up to 24 months	ORR, 15% (PD-L1+, 20%; PD-L1-, 7.9%; SCC, 17.6%; AC, 12.5%)
(34)	CheckMate 358 NCT02488759 (Multi-location)	I/II	19	SCC = 100%	R/M CC; SCC; HPV+	Nivolumab 240 mg IV q2w for up to 2 years	ORR, 26.3%
(35)	Tamura et al., 2019 (Japan)	II	20	SCC = 70% AC = 25% ASC = 5%	Advanced or recurrent CC; ≥1 lines previous chemotherapy regimen	Nivolumab 240 mg IV q2w until CR or PD, unacceptable toxicity, investigator decision, or withdrawal of consent	ORR, 25% (PD-L1+, 33%; PD-L1-, 0%)
(32)	NRG-GY002 NCT02257528 (Multi-location)	II	25	SCC = 60.0% AC = 24.0% ASC = 16.0%	Persistent R/M CC; ≥1 lines prior systemic chemotherapeutic regimen	Nivolumab 3mg/kg IV q2w for a maximum of 46 doses until PD or adverse effects prohibit therapy.	ORR, 4%
(42)	Lheureux et al., 2018 (U.S. and Canada)	I/II	42	SCC = 69% AC = 31%	R/M CC	The run-in cohort (phase 1): Ipilimumab, 3mg/kg IV q3w for 4 cycles; The second cohort (phase 2): Ipilimumab, 10mg/kg IV q3w for 4 cycles followed by 10mg/kg IV q12w for 4 additional cycles as maintenance therapy for pts with radiologic response or stabilization	ORR, 2.4%; Safety: AEs ≥ Grade 3, 12 pts
(43, 44)	NCT03676959 (China)	I	Dose-escalation phase = 12 Dose-expansion phase = 92	SCC = 95.7% AC = 2.1% ASC = 1.1% Other = 1.1%	R/M CC; previously failed or intolerant to the first-line platinum-based regimen	Socazolimab 5mg/kg IV q2w until PD	ORR, 15.4% (PD-L1+, 16.7%; PD-L1-, 17.9%); Safety: MTD was not determined at the highest dose (15 mg/kg); No treatment-related deaths
(45)	AK104-201 Wu et al., 2022 (China)	II	111	SCC = 92.8%	R/M CC; progressed on or after two or fewer previous doublet chemotherapy with or without bevacizumab	Cadonilimab 6 mg/kg IV q2w	ORR, 33.0% (PD-L1+, 43.8%)
(46, 47)	NCT03427411 (U.S.)	II	59	CC = 55.9%	Locally advanced or metastatic HPV associated malignancies including: Non-Neuroendocrine CCs	Bintrafusp alfa 1200mg IV q2w until PD, unacceptable toxicity, or study withdrawal	ORR, 30.5%
(38)	NCT05171790 (China)	Ib	53	SCC = 83% AC = 17%	R/M CC	QL1706 5.0 mg/kg IV q3w for up to 24 months	ORR, 28%

R/M, recurrent or metastatic; CC, cervical cancer; SCC, squamous cell carcinoma; AC, adenocarcinoma; ASC, adenosquamous carcinoma; NEC, neuroendocrine cell carcinoma; GCC, Glassy cell carcinoma; BSCC, basaloid squamous cell carcinoma; ECOG, Eastern Cooperative Oncology Group performance status; HPV, human papillomavirus; IV, intravenous; ORR, objective response rate; OS, overall survival; PFS, progression-free survival; CR, complete response; PD, progressive disease; AEs, adverse events; pts, patients; MTD, maximum tolerated dose; CPS, combined positive score; q2w/q3w/q12w, once every two/three/twelve weeks.

### 3.1 Anti-PD-1 inhibitors

#### 3.1.1 Pembrolizumab (MK-3475)

A phase 2 basket clinical trial (KEYNOTE-158/NCT02628067) was conducted to assess the use of Pembrolizumab for the treatment of 98 patients with advanced CC, of whom 77 were R/M after prior chemotherapy. This trial had a 12.2% objective response rate (ORR), including three patients with complete responses (CRs) and nine with partial responses (PRs) (33). A subsequent Korean multicenter retrospective real-world study (KGOG1041) assessed pembrolizumab monotherapy in 117 patients with R/M CC, of whom three had CRs and eight had PRs for an ORR of 9.4%. Two patients had suspected treatment-related deaths, and eight (6.8%) had grade 3 or higher treatment-related adverse events (TRAEs) (39). These results suggested that Pembrolizumab may prolong the survival of patients with advanced or R/M CC. Better outcomes may be expected from combination therapy; however, particular attention will need to be placed on cumulative toxicity.

#### 3.1.2 Nivolumab

Results from a phase I/II clinical trial (CheckMate 358/NCT02488759) found that the single-agent, Nivolumab, achieved an ORR of 26.3% and a median OS of 21.9 months among patients with recurrent HPV-positive CC, with 12 of 19 (63.2%) experiencing TRAEs (34). In another multicenter phase II study in Japan, the ORR of patients with advanced or recurrent CC after first-line chemotherapy who received single-agent Nivolumab was 25%. Patients in the PD-L1+ group had an ORR of 33%, while those in the PD-L1- group had no response (35). Patients in a subsequent phase II clinical trial (NRG-GY002/NCT02257528) had an ORR of only 4%, with low single agent activity (32). Second-line Nivolumab treatment is still in clinical trials with widely diverse results, and its performance in combination therapy is expected.

#### 3.1.3 Cemiplimab (REGN2810)

In R/M squamous CC, Cemiplimab alone or in combination with hypofractionated radiotherapy was effective in a phase I clinical study (NCT02383212) (48). The findings of an open-label, multi-center, randomized controlled phase III clinical study (NCT03257267) were recently published. Single-agent chemotherapy with Pemetrexed, Topotecan, Irinotecan, Gemcitabine, or Vinorelbine, was chosen by the investigator for use as controls. Patients in the Cemiplimab group had a median OS of 12.0 months, 3.5 months longer than the OS of the 608 patients in the chemotherapy group. This treatment benefited patients with both squamous and adenocarcinoma for an ORR of 16.4%. The incidence of grade ≥3 TRAEs was also less in the Cemiplimab group than in the chemotherapy group (45.0% versus 53.4%, respectively) (40). This provided a new option for R/M CC to serve as a second-line treatment (49).

#### 3.1.4 Balstilimab (AGEN2034)

An open-label, single-arm phase II clinical study (NCT03104699) was conducted to examine the efficacy of Balstilimab among patients with R/M CC after receiving first-line platinum-based chemotherapy. Preliminary findings revealed an ORR of 15% (including five patients with CRs and 16 with PRs), a median response time of 15.4 months among 140 evaluable patients, and a disease control rate (DCR) of 49.3%. PD-L1-positive patients CPS ≥ 1% had an ORR of 20%, while PD-L1-negative patients had an ORR of 7.9%. The ORR was 12.5% among patients with cervical adenocarcinoma, demonstrating that these responses were not restricted to patients with a pathological type of squamous carcinoma. The most prevalent grade 3 or higher TRAEs were immune-mediated enterocolitis (3.1%) and diarrhea (1.9%), suggesting a manageable safety profile (41).

### 3.2 Anti-CTLA-4 inhibitors

#### 3.2.1 Ipilimumab

A phase 1/phase 2 single-arm, multi-center clinical trial from the United States and Canada assessed the safety and anticancer efficacy of Ipilimumab [an anti-CTLA-4 monoclonal antibody (mAb)], which was used as a single-drug treatment for HPV-associated R/M CC. Ipilimumab monotherapy was well tolerated by patients but lacked significant single-agent activity. However, multicolor flow cytometry of peripheral lymphocytes revealed that levels of human leukocyte antigen-antigen D-related, inducible T-cell costimulator (ICOS), and PD-1 increased after the initial course of treatment and returned to baseline during maintenance therapy, which could guide further clinical trials to explore combination dosing options (42).

### 3.3 Anti-PD-L1 inhibitors

#### 3.3.1 Socazolimab (ZKAB001)

A phase I clinical study (NCT03676959) of Socazolimab among R/M CC patients in China was presented at the 2022 ASCO meeting. There were 92 patients who entered the 5 mg/kg dose-expansion phase. The median PFS was 4.44 months, the median OS was 14.72 months, and the ORRs were similar between PD-L1 positive and negative patients (16.7% and 17.9%, respectively). Only 7.7% of patients had grade 3–4 TRAEs. These findings demonstrated the safety and efficacy of single-agent Socazolimab for R/M CC treatment (43, 44).

### 3.4 Bispecific antibody

#### 3.4.1 Cadonilimab (AK104)

Cadonilimab is a PD-1/CTLA-4 bi-specific antibody. A multi-center, open-label, single-arm phase II study from China enrolled 111 patients with R/M CC who had previously received up to two lines of doublet chemotherapy with or without Bevacizumab [a humanized IgG1 anti-vascular endothelial growth factor (VEGF) mAb]. The results were presented by Wu et al. at the SGO Annual Meeting in March 2022 and revealed that the ORR of Cadonilimab monotherapy was 33.0% (including 12 patients with CRs and 21 with PRs), the OS was 17.51 months, and the median PFS was 3.75 months. In PD-L1-positive (CPS ≥1) patients, the ORR was 43.8%, the median PFS was 6.34 months, and the median OS was not reached. Grade ≥3 TRAEs occurred in 28.8% of patients, with the most common being anemia (7.2%) and decreased appetite (2.7%) (45). In June 2022, Cadonilimab was authorized in China for the treatment of R/M CC patients who progressed during or following platinum-based chemotherapy (50).

#### 3.4.2 Bintrafusp alfa (M7824/MSB0011359C)

Bintrafusp alfa is an innovative bifunctional fusion protein consisting of the extracellular domain of transforming growth factor-β receptor II (TGF-βRII) fused to human IgG1 mAb of PD-L1 (51). Strauss et al. used the results of a phase 1 clinical study to retrospectively analyze the efficacy of Bintrafusp alfa in the treatment of HPV-associated malignancies. Findings from the phase 1 and phase 2 clinical trials (NCT02517398 and NCT03427411) of 59 patients revealed an ORR of 30.5% (five patients with CRs) and a DCR of 44.1% [eight patients with stable disease (SDs)], suggesting that this protein is worth further exploration for the treatment of CC (46, 52).

## 4 Combination therapy for R/M CC

Given the limited efficacy of ICB monotherapies, studies have begun to assess combination therapies such as ICB combination therapy, ICB and targeted therapy, radiotherapy/chemotherapy, antibody-drug conjugate (ADC), adoptive cell therapy, and therapeutic vaccines. These include the use of new drugs alone or in combination with classical therapeutic regimens, such as the anti-PD-L1 mAb, Tislelizumab, in combination with the anti-TIGIT mAb, Ociperlimab, in a phase II study (AdvanTIG-202) (53). While chemotherapy can kill tumor cells, causing the release of tumor-associated antigens and promote tumor cell recognition by T cells, immunotherapy can directly activate T cells. While anti-angiogenic therapy and radiotherapy regulate the immunosuppressive microenvironment, immunotherapy increases the sensitivity of radiotherapy. Indeed, the combination of an activated immune response and multiple therapies can have a synergistic anti-tumor effect (54–56). On October 13, 2021, Pembrolizumab in combination with chemotherapy, with or without Bevacizumab, was approved by the U.S. FDA for first-line treatment of R/M CC with CPS ≥1. Other treatment options remain in the clinical trial phase, most as second-line therapies. Published data on ICB in combination with other treatments for R/M CC is shown in **Table 3** (19, 57–70).

**Table 3:** Published data on combination therapy with immune checkpoint drugs for recurrent/metastatic cervical cancer.

Ref.	Trial	Phase	N	Histology	Patient population	Treatment regimens	Primary endpoint
(19, 57)	KEYNOTE-826 NCT03635567 (Multi-location)	III	Pembrolizumab Group = 308 Placebo Group = 309	Pembrolizumab Group: SCC = 76.3% AC = 18.2% ASC = 4.9%; Placebo Group: SCC = 68.3% AC = 27.2% ASC = 4.5%	R/M CC; PD-L1-positive (CPS ≥ 1); not treated with systemic chemotherapy and not amenable to curative treatment	Pembrolizumab200mg IV or placebo q3w for up to 35 cycles and Paclitaxel 175 mg/m2 IV + Cisplatin 50 mg/m2 IV or Carboplatin AUC 5 IV, with or without Bevacizumab 15mg/kg IV (investigator’s choice)	Pembrolizumab vs. Placebo: median PFS, (CPS ≥1) 10.4 months vs. 8.2 months; median PFS, (CPS ≥10) 10.4 months vs. 8.1 months; 24 months OS, 53.0% vs, 41.7%
(58)	NCT04868708 (China)	II	45 (3 cohorts: A-15, A-10, and B-10)	NR	R/M CC; SCC, AC, or ASC	A-15/A-10: Cadonilimab (AK104) 15/10 mg/kg IV + Paclitaxel 175mg/m2 IV + Cisplatin 50 mg/m2 IV/Carboplatin AUC 5 IV, q3w; B-10: AK104 10 mg/kg IV + Paclitaxel 175 mg/m2 IV + Cisplatin 50 mg/m2 IV/Carboplatin AUC 5 IV+ Bevacizumab 15 mg/kg IV, q3w). All pts received AK104 until PD or unacceptable toxicity	Safety: AEs ≥ Grade 3, 23 (51.1%) pts
(59)	Lan et al., 2022 (China)	II	45	SCC = 66.7% AC = 33.3%	R/M CC; progressed after at least one line of systemic therapy	Camrelizumab 200mg IV q2w and Apatinib 250mg orally qd for up to 24 months	ORR, 55.6%
(60)	Xu et al., 2022 (China)	II	42	SCC = 83.3% AC = 11.9% ASC = 4.8%	R/M CC; PD-L1-positive (CPS ≥ 1); previously failed or intolerant to the first-line systemic therapy	Sintilimab 200mg IV q3W and Anlotinib 10mg orally qd on days 1-14 per cycle.	ORR, 54.8%
(61)	Cheng et al., 2022 (China)	Retrospective	102 (Camrelizumab = 85; Sintilimab = 27)	SCC = 73.5% AC = 26.5%	R/M CC	Camrelizumab/Sintilimab 200mg IV q3w + Paclitaxel (175 mg/m2) and Cisplatin (50 mg/m2)/Carboplatin (AUC = 5) q3w ± Apatinib 250mg orally qd for up to 2 years	ORR, 51.0%; median PFS, 11.0 months
(62)	ENGOT-GYN3/AGO/LIO; NCT04042116 (Multi-location)	II	20	NR	R/M CC; ≥1 lines prior platinum-based chemotherapy regimen ± Bevacizumab	Nivolumab 480 mg IV q4w + Lucitanib 6mg orally qd	ORR, 23.5%
(63)	NCT04150575 (China)	II	21	NR	CC; PD-L1-positive (CPS ≥ 1); previously failed or intolerant to the first-line systemic therapy	Serplulimab 4.5 mg/kg + Albumin-bound paclitaxel 260 mg/m2 q3w	ORR, 57.1%; Safety: The most common AEs ≥ Grade 3, decreased neutrophil count, 7 (33.3%) pts
(64)	NCT02921269 (U.S.)	II	11	SCC = 55% AC = 45%	Persistent R/M CC; progression after 1–2 prior therapies	Atezolizumab 1200mg IV q3w + Bevacizumab 15mg/kg IV q3w until PD or unacceptable toxicity	ORR, 0%
(65)	PRIMMO NCT03192059 (Belgium)	II	18	SCC = 66.7% AC = 27.8% ASC = 5.6%	Persistent R/M CC; Progression ≥1 lines of standard chemotherapy	Induction period: the immunomodulatory five-drug cocktail [Cyclophosphamide 50mg, Aspirin 325mg, Lansoprazole 180 mg or 30 mg (dose alternating weekly), Vitamin D 50μg, and Turmeric hytosome 2g] orally qd for 2 weeks; Pembrolizumab 200mg IV q3w for up to 2 years; SBRT (24Gy/3 fractions) over five days during the first cycle of Pembrolizumab (study days 15, 17, and 19).	ORR, 11.1%
(66)	NCT03495882 (Multi-location)	II	155	SCC = 70.3% AC = 27.1% ASC = 2.6%	R/M CC; relapsed after ≥1 lines prior platinum-based chemotherapy regimen	Balstilimab 3mg/kg IV q2w and Zalifrelimab 1mg/kg IV q6w for up to 24 months.	ORR, 25.6% (PD-L1+, 32.8%; PD-L1-, 9.1%)
(67)	ENGOT Cx8/GOG 3024/ innovaTV 205; NCT03786081 (Multi-location)	II	32	NR	R/M CC	Tisotumab Vedotin 2mg/kg IV and Pembrolizumab 200mg IV q3w	ORR, 41%
(68, 69)	KEYNOTE-567 NCT03444376 (South Korean)	II	36	SCC = 78% AC = 22%	Recurrent/Advanced HPV-Positive (HPV-16/HPV-18) CC	GX-188E 2mg IM at weeks 1, 2, 4, 7, 13, and 19, with one optional dose at week 46 that was at the investigator’s discretion, and Pembrolizumab 200mg IV q3w for up to 2 years	ORR within 24 weeks, 42%
(70)	Yin et al., 2020 (China)	Retrospective	80	SCC = 81.25% AC = 18.75%	Metastatic CC with low MSI expression and PDL1-negative	Nivolumab 3mg/kg IV q2w until PD or unacceptable toxicity and TILs transfused at the first cycles	Safety: The most common AEs ≥ Grade 3, Fever, 4 (5%) pts

R/M, recurrent or metastatic; CC, cervical cancer; SCC, squamous cell carcinoma; AC, adenocarcinoma; ASC, adenosquamous carcinoma; HPV, human papillomavirus; IV, intravenous; IM, intramuscular; ORR, objective response rate; OS, overall survival; PFS, progression-free survival; PD, progressive disease; AEs, adverse events; pts, patients; MTD, maximum tolerated dose; CPS, combined positive score; q2w/q3w/q4w/q6w, once every two/three/four/six weeks; qd, once daily; NR, Not Reported; SBRT, Stereotactic body radiotherapy; MSI, microsatellite instability; TIL, tumor-infiltrating lymphocyte.

### 4.1 Combination of ICB and systemic therapy

#### 4.1.1 Pembrolizumab ± bevacizumab

A double-blind, randomized, phase III clinical trial (NCT03635567/KEYNOTE-826) combining Pembrolizumab with chemotherapy ± Bevacizumab enrolled PD-L1-positive (CPS ≥1) metastatic or unresectable CC patients that showed progress during chemotherapy. Of 548 PD-L1-positive patients, the median PFS in the Pembrolizumab and placebo groups was 10.4 and 8.2 months, respectively, and the 1-year OS rates were 53.0% and 41.7%, respectively. Anemia (Pembrolizumab group: 30.3%; placebo group: 26.9%) and neutropenia (Pembrolizumab group: 12.4%; placebo group: 9.7%) were the most common grade 3–5 TRAEs (19). Thus, 2022 NCCN recommendations for first-line treatment of patients with PD-L1-positive (CPS ≥1) R/M CC advocate for the use of Pembrolizumab in combination with platinum or paclitaxel, with or without Bevacizumab (71). At the 2022 ASCO meeting, Tewari et al. added the results of a subgroup analysis using a Cox regression model, in which patients in the Pembrolizumab group had better survival rates than those in the placebo group among the subgroups of bevacizumab use, platinum use, prior CRT, and squamous carcinoma (57).

#### 4.1.2 Cadonilimab ± bevacizumab

Results of a phase II study in China that combined Cadonilimab with platinum-based chemotherapy ± Bevacizumab (bev) were presented at the 2022 ASCO meeting, including three cohorts (A-15: AK104 15 mg/kg; A-10: AK104 10 mg/kg; B-10: AK104 10 mg/kg + bev 15 mg/kg). The ORRs were 73.3% (11/15), 68.8% (11/16), and 92.3% (12/13), respectively, and were significant regardless of CPS status. Twenty-three (51.1%) patients had grade ≥3 TRAEs and eight (17.8%) had grade ≥3 immune-related adverse events (irAE). There were no deaths associated with AK104 (58). These data suggest that AK104 plus chemotherapy may be a first-line treatment option for R/M CC. Additional phase III study findings are needed to confirm this preliminary result.

#### 4.1.3 Camrelizumab + apatinib

The antitumor efficacy and safety of Camrelizumab (a humanized anti-PD-1 mAb) in combination with Apatinib [a vascular endothelial growth factor receptor-2 (VEGFR-2) inhibitor] for the treatment of R/M CC was studied in an open-label, multicenter, single-arm, phase II clinical study in China. The intention-to-treat analysis results revealed an ORR of 55.6% (25/45), a CR of 4.4% (2/45), and a PR of 51.1% (23/45), with the combination therapy showing better outcomes than the monotherapy. However, only 35.6% of patients were able to tolerate the starting dose of 250 mg once daily Apatinib, and the combination led to increased adverse events (AEs). While the optimal dose-response relationship requires further study, this combination is likely to be an effective option for the treatment of advanced CC (59).

#### 4.1.4 Sintilimab + anlotinib

Sintilimab is a humanized IgG4 anti-PD-1 mAb (72). Anlotinib (AL3818) was a small molecule VEGFR-2 selective inhibitor (73). A phase II, single-arm, multicenter clinical study in China found that 39 patients with R/M CC and evaluable efficacy of PD-L1-positive (CPS ≥1) had an ORR of 59.0%, a median PFS of 9.4 months, and a DCR was 94.9%. The higher ORR was associated with mutations in the PIK3CA, KMT2D, or PI3K-AKT signaling pathways, while the shorter PFS was linked to STK11 and/or JAK2 mutations (60).

#### 4.1.5 Sintilimab/camrelizumab ± apatinib

A retrospective study of 102 patients with R/M CC who received treatment with the PD-1 mAb (Sintilimab/Camrelizumab) in combination with or without anti-angiogenic medicines (Apatinib) in China showed an ORR of 51.0%, a DCR of 66.7%, and a median PFS of 11.0 months. This study found that the combination of PD-1 blockade of chemotherapy and anti-angiogenic medicines successfully extended the ORR and PFS of patients with R/M CC, especially among those with squamous carcinoma, with a recurrence time >6 months (61).

#### 4.1.6 Nivolumab + lucitanib

The anti-PD-1 mAb, Nivolumab, in combination with Lucitanib, a tyrosinase inhibitor with multiple targets (VEGFR1-3, FGFR1-3, and PDGFRα/β) was designed as a novel treatment for R/M CC. Partial findings of a phase II study (LIO-1) were reported at the 2022 SGO Annual Meeting. Seventeen of 20 enrolled patients underwent post-treatment evaluation, with four having PRs and eight showing SDs. Hypertension (20%) was the most prevalent grade ≥3 treatment-emergent adverse event (TEAE). Three patients stopped using lucitanib because of associated TEAEs, including colonic fistula, hypertension, and proteinuria (62). The combination had strong anti-tumor activity and more results are expected soon.

#### 4.1.7 Serplulimab + albumin-bound paclitaxel

The findings of a multi-center, single-arm phase II study (NCT04150575) in China that combined Serplulimab, an anti-PD-1 mAb, with albumin-bound paclitaxel among patients with advanced CC who relapsed, progressed, or were unable to tolerate first-line chemotherapy were reported at the 2022 SGO Annual Meeting. The study enrolled 21 patients with PD-L1-positive (CPS ≥1) advanced CC. The ORR was 57.1% (three patients with CRs and nine with PRs), the DCR was 76.2%, and the median duration of response (DOR) was not reached, with a median follow-up time of 14.6 months. The median PFS was 5.7 and the OS was 15.5 months, which were significantly better than monotherapy. The safety profile was good, with grade ≥3 TEAEs dominated by hematologic toxicities such as decreased neutrophil count (33.3%), decreased white blood cell count (28.6%), and anemia (23.8%), and the absence of grade 4–5 irAEs (63). These findings suggest that this combination may serve as a good option for the second-line treatment of R/M CC.

#### 4.1.8 Atezolizumab + bevacizumab

Bevacizumab in combination with atezolizumab failed to provide the predicted effectiveness endpoints in a phase II clinical trial (NCT02921269) and should be used with caution in clinical practice (64). A further phase III randomized controlled BEATcc trial (NCT03556839) is ongoing, with results expected in 2023 (74).

### 4.2 Combination of ICB and radiotherapy/chemotherapy

#### 4.2.1 Pembrolizumab + radiotherapy

Despite ICB’s partial efficacy against certain CC subtypes, single-agent ICB has limited efficacy in patients with R/M CC due to the tumor suppressive microenvironment, and most new ICB drugs are expensive. A multi-center, open-label, non-randomized phase II clinical trial (NCT03192059) in Belgium designed a regimen that combines existing drugs by applying Pembrolizumab (200 mg in 21-day treatment cycles), radiotherapy (3x8Gy in 48 h-intervals), and a regimen consisting of aspirin, lansoprazole, vitamin D, cyclophosphamide and curcumin in an immunomodulatory cocktail treatment (PRIMMO) that simultaneously acts on tumor metabolism, angiogenesis, and anti-tumor immune activity (75). Jaeghere et al. found that in 18 patients with R/M CC, the ORR was 11.1%, the median interval-censored PFS was 4.1 weeks, and the median DOR was not reached. Grade ≥3 TRAEs were reported in 10 (55.6%) patients (65). This immunomodulatory five-drug cocktail therapy is a new treatment with durable but limited antitumor activity. However, its toxicity requires further study, and the results from a larger sample size will help to verify drug efficacy.

### 4.3 ICB combination therapy

#### 4.3.1 Balstilimab + zalifrelimab

Zalifrelimab (AGEN1884) is a humanized IgG1 anti-CTLA-4 mAb (76). A recent phase II study (NCT03495882) found an ORR of 25.6% in 125 patients with measurable R/M CC who were treated with Balstilimab (Bal) in combination with Zalifrelimab (Zal), including 10 patients with CRs and 22 with PRs. The ORR was 32.8% and 9.1% in patients with positive (CPS ≥1%) and negative (CPS <1%) PD-L1 status, respectively (66). The addition of Bal to Zal increased the ORR and DOR without significant TRAEs (77). Another phase II trial, RaPiDS (GOG-3028/NCT03894215), is also ongoing (78).

#### 4.3.2 Navoximod + atezolizumab

In a phase I clinical trial of Navoximod (GDC-0919), an IDOi, in combination with the PD-L1 blocker, Atezolizumab, for the treatment of patients with advanced solid tumors, clinical efficacy was demonstrated at all dosage levels in all tumor types, including CC, with tolerable AEs. However, there was no direct evidence that the combination therapy was superior to monotherapy, matching findings from another clinical trial (ECHO-301) (79).

### 4.4 Others

#### 4.4.1 Pembrolizumab + tisotumab vedotin

Tisotumab vedotin (TV) is an ADC that targets tissue factors and releases a microtubule-disrupting agent, monomethyl auristatin E (80). A multi-center, phase 1/2 clinical study, InnovaTV 205/ENGOT-cx8 (NCT03786081/GOG-3024), investigated the effectiveness and safety of TV in conjunction with Pembrolizumab as a first-line therapy for R/M CC (81). In the latest interim analysis reported by Dr. Domenica Lorusso at the 2022 ASCO meeting, the ORR was 41% (three patients had CRs and 10 had PRs) among 32 evaluable patients. The median time to response was 1.4 months, the median PFS was 5.3 months, and the median DOR and OS were not reached. A report of the final outcomes is expected (67).

#### 4.4.2 Pembrolizumab + GX-188E

GX-188E (Tirvalimogene teraplasmid) is an HPV-16 and HPV-18 E6 and E7 therapeutic DNA vaccine (82). Pembrolizumab in conjunction with GX-188E in patients with HPV16/18-positive R/M CC was tested in a single-arm phase II clinical trial (NCT03444376) from South Korea. The ORR was 42% with four patients with CRs, seven with PRs, and four (11%) with grade 3-4 AEs at interim analysis (68). The findings of the subsequent KEYNOTE-567 trial were reported at the 2021 ASCO Annual Meeting and showed an ORR of 33.3% (16/48) following combination therapy, a significant improvement over monotherapy. A higher ORR was found in PD-L1-positive, HPV16-positive, squamous cancer patients, with a safety profile comparable to monotherapy (69).

#### 4.4.3 Nivolumab + tumor-infiltrating lymphocytes (TILs)

Nivolumab is a humanized IgG4 anti-PD-1 mAb (83). Yin et al. showed that patients with low microsatellite instability (MSI) expression, PD-L1 negative metastatic CC who were treated with Nivolumab in combination with TILs had an ORR of 25%, a median PFS of 6.1 months, and a median OS of 11.3 months. This provided a new treatment option for patients who are unresponsive to most immunotherapies (70).

## Combination of ICB and radiotherapy/chemotherapy for LACC

Since CCRT is the standard of care for LACC, current studies have focused on immunotherapy combined with CCRT. Immunotherapy can increase the sensitivity of radiotherapy and further improve the local control rate while activating the immune system, helping to eliminate subclinical lesions and reduce recurrence. All published data on ICBs for LACC are in early-stage clinical trials. Moreover, the multi-center CALLA study of Durvalumab (NCT03830866) and the DECISION study in Japan (jRCT2031210083) are ongoing. The CALLA study is a large phase III clinical study that is expected to end in April 2024 (84, 85). A comparison of current published data on LACC treatment options is shown in **Table 4** (86–91).

**Table 4:** Published data on combination therapy with immune checkpoint drugs for locally advanced cervical cancer.

Ref.	Trial	Phase	N	Histology	Patient population	Treatment regimens	Primary endpoint
(86)	NCT02635360 (U.S.)	II	52	SCC = 82.7% AC = 15.4% ASC = 1.9%	FIGO 2009 stages IB-IVA CC	Pembrolizumab 200mg IV q3w for 3 cycles as consolidative (Arm 1) or concurrent (Arm2) therapy in addition to CRT (EBRT 45 Gy with Cisplatin 40mg/m2 q1w for 5-6 weeks, followed by a brachytherapy boost to ≥ 80 Gy)	Safety: AEs ≥ Grade 3, 23 (44.2%) pts; AEs ≥ Grade 4, 11 (21.2%) pts; No AEs ≥ Grade 5
(87)	NiCOL NCT03298893 (France)	I	16	SCC = 87.5% AC = 12.5%	FIGO 2018 stage IB3-IVA CC	Nivolumab 240mg IV q2w for up to 6 months as concurrent and maintenance therapy in addition to CRT (45 Gy in 25 fractions with cisplatin 40mg/m2 q1w, followed by a brachytherapy boost to 85 Gy)	Safety: DLT, 3 pts; AEs ≥ Grade 3, hypotension (2 pts) and acute kidney injury (1 pts)
(88)	GOTIC-018/JMA-IIA00425 (Japan)	I	30	SCC = 93.3% AC = 6.7%	FIGO 2009 stages IB-IVA CC	Cohort A: Nivolumab 240mg IV q2w with CRT followed by maintenance therapy with Nivolumab; Cohort B: pre- (two doses of Nivolumab before CCRT) plus Cohort A; CCRT: ≥ 4 cycles of Cisplatin (40 mg/m2 q1w) and EBRT followed by brachytherapy	Safety: AEs ≥ Grade 3, all pts [neutropenia (60.0 and 26.7% in cohort A and B, respectively), anemia (13.3 and 16.7%) and diarrhea (13.3 and 26.7%)]
(89)	ChiCTR2000032879 (China)	I	22	NR	FIGO stages II-IVA CC	Toripalimab 240mg IV on days 1, 22 and 43; CRT: Cisplatin 40mg/m2 q1w for 5 weeks and EBRT 45-50.4Gy, followed by brachytherapy 24-30Gy	Safety: AEs ≥ Grade 3, 10 (45.5%) pts
(90)	GOG-9929 NCT01711515 (U.S.)	I	32	SCC = 88% AC = 9% ASC = 3%	CC, FIGO stages IB2 or IIA with positive PALNs or stages IB, IIIB, or IVA with positive pelvic lymph nodes, PALNs, or both	Cisplatin 40mg/m2 for 6 weeks concurrent with Extended field RT 45Gy followed by a brachytherapy (LDR 40Gy; HDR 30Gy); Ipilimumab (3mg/kg or 10mg/kg) IV q3w for 4 doses	Safety: AEs ≥ Grade 3, 21 (9.5%) pts (lipase increase; dermatitis)
(91)	NRG GY-17; NCT03738228 (U.S.)	I/Ib	36	NR	LACC with positive lymph nodes	Three doses of Atezolizumab (1200mg IV) on day -21, 0, 21 (Arm A) versus day 0, 21, 42 (Arm B) and CRT	Safety: DLT, Arm A: no DLTs; Arm B: 3 (8%) pts; AEs ≥ Grade 3, Arm A: 3 (8.3%) pts; Arm B: 7 (19.4%) pts

CC, cervical cancer; LACC, locally advanced cervical cancer; FIGO, the International Federation of Gynecology and Obstetrics; SCC, squamous cell carcinoma; AC, adenocarcinoma; ASC, adenosquamous carcinoma; IV, intravenous; AEs, adverse events; pts, patients; DLT, dose-limiting toxicities; q1w/q2w/q3w, once every one/two/three weeks; NR, Not Reported; SBRT, Stereotactic body radiotherapy; EBRT, external-beam radiation therapy; CCRT, concurrent chemoradiotherapy; PALN, para-aortic lymph node.

### 5.1 Anti-PD-1 inhibitors

#### 5.1.1 Pembrolizumab

A randomized phase II study (NCT02635360) in U.S. showed that Pembrolizumab was safe after or during CCRT among patients with LACC. CCRT was cisplatin plus pelvic radiotherapy. Of 52 patients with evaluable toxicity, at least grade 4 AEs were observed in 1/11 patients, grade 3 AEs were found in 1/23 patients, two patients experienced three dose-limiting toxicities (DLTs), and 83% of patients completed Pembrolizumab treatment. These results are promising (86).

#### 5.1.2 Nivolumab (ONO-4538)

Results of two phase I studies of Nivolumab in combination with CCRT for patients with LACC were reported at the 2022 ASCO meeting. The NiCOL trial (NCT03298893) in France, a prospective, multi-center, dose-confirmation, phase I clinical trial, enrolled 16 patients with stage IB3-IVA CC receiving Nivolumab (240 mg once every 2 weeks) during CCRT and Nivolumab maintenance therapy following CCRT. The 1-year PFS was 81.2% and three cases experienced DLT, including two with grade 3 hypotension and one with grade 3 acute kidney injury (87). Another multi-center GOTIC-018 study in Japan compared individuals receiving Nivolumab during CCRT followed by Nivolumab maintenance therapy (Group A) with those receiving Nivolumab before, during, and after CCRT (Group B). The most common grade ≥3 AEs were neutropenia (Groups A: 60.0%; Group B: 26.7%), anemia (Groups A: 13.3%; Group B: 16.7%), and diarrhea (Groups A: 13.3%; Group B: 26.7%), and no DLT was found during the acute phase (88). Overall, the combination of Nivolumab and CCRT was shown to be safe and effective at improving PFS, and further trial results are expected.

#### 5.1.3 Toripalimab

Patients with stage III-IVA CC were enrolled in an open-label, single-arm, phase II study (NCT05084677) in China to evaluate ORR, PFS, OS, and safety. Toripalimab (240 mg, once every 3 weeks) was administered during CCRT and consolidation chemotherapy, and Toripalimab (240 mg, once every 6 weeks) was administered after consolidation chemotherapy until the entire treatment cycle reached one year (92). At the 2022 ASCO meeting, Ou et al. presented the results of 22 LACC patients receiving brachytherapy and Toripalimab (240 mg on days 1, 22, and 43) after CCRT. The 3-month ORR rate was 95.5%. However, two patients developed multiple metastases and lung metastasis after 3 and 6 months of treatment, respectively. The most common grade III AE was leukopenia (36.4%), and the most common irAE was hypothyroidism (9.1%) (89). Toripalimab in combination with CCRT is promising for the treatment of patients with LACC, and further follow-up results are expected.

### 5.2 Anti-CTLA-4 inhibitors

#### 5.2.1 Ipilimumab

A prospective phase 1 clinical trial (GOG 9929/NCT01711515) in U.S. investigating the combination immunotherapy regimen of sequential Ipilimumab after CCRT for patients with metastases in the pelvic lymph nodes, para-aortic lymph nodes, or both had a PFS of 81% and an OS of 90% during the first year, with better long-term outcomes than expected from the GOG0125 study (90). New results from the GOG-9929 study showed that Ipilimumab increased T-cell activation, including ICOS and PD-1 expression, and maintained CD4+ T-cell levels among patients at high risk of recurrence. These data suggest that combination therapy may further boost the antitumor immune response (93).

### 5.3 Anti-PD-L1 inhibitors

#### 5.3.1 Atezolizumab

A phase I clinical study (NRG-GY017/NCT03738228) in U.S. evaluated the immune activation of Atezolizumab (an anti-PD-L1 mAb) in lymph node-positive patients with LACC who received extended-field chemoradiotherapy (94). Preliminary results were reported at the 2022 SGO Annual Meeting, where on-treatment biopsy results showed that patients with higher pre-treatment T-cell receptor (TCR) diversity were more likely to experience pathologic CR and that the drug combination had a good safety profile (91).

## 6 Biomarkers for predicting the efficacy of immunotherapy

There are no highly predictive biomarkers for immunotherapy of R/M CC. The most used is PD-L1 expression and several clinical studies, including KEYNOTE-158 and NCT03104699, have shown that patients with CPS ≥1 (PD-L1 positive) have better outcomes than those with CPS <1 (PD-L1 negative) (33, 41). Patients with CPS ≥1 were subsequently included in the KEYNOTE-826 trial and used as an indication for Pembrolizumab by the NCCN guidelines. However, the NCT03676959 trial did not observe a difference in treatment efficacy between the PD-L1 positive and negative groups (43, 44). Whether PD-L1 expression levels can be used as a marker remains controversial and varies widely across tumor types. Among all FDA-approved immune checkpoint inhibitors, PD-L1 was predictive in only 28.9% of cases (95). More large multicenter clinical trials are needed to validate the initial findings. In addition, CC is an HPV-associated tumor, and PD-L1 positivity is often associated with a poorer prognosis (96). Further exploration of the mechanism of interaction between HPV infection and PD-L1 expression may help explain the difference in treatment efficacy.

A retrospective analysis evaluated the association between prognosis and tumor mutational burden (TMB) among 151 patients with different tumor types receiving multiple single-agent immunotherapies, and multivariate analysis found that a TMB of ≥20 mutations per million bases (≥ 20 mut/Mb) suggested a better prognosis and that TMB was an independent prognostic factor in patients receiving ICB monotherapy (97). However, this finding was not observed in combination therapy and requires further validation in prospective studies, as reaffirmed by Chan et al. (98). Some studies showed that patients treated with PIK3CA, PTEN gene mutation, and TMB-high (≥5 mut/Mb), had a better prognosis, while those with an ERBB3 gene mutation had a poor prognosis, suggesting that these could serve as predictive biomarkers for combination therapy (99). In addition, MSI, CD8+ tumor-infiltrating lymphocytes (CTLs)/CD4+ regulatory T cells (Treg) ratio (positively correlated with prognosis), mismatch repair (MMR), polymerase δ and ϵ mutations were also potential predictive biomarkers (100–102). Alterations in the 9p24.1 gene copy number and the density of PD-1 positive DCs could be used to screen appropriate populations for PD-1/PD-L1 blockade therapy (103, 104).

Current studies suggest that immunotherapy using a combination of multiple biomarkers promotes a more effective anti-tumor response than individual markers (105). Well-defined markers can help screen patients most likely to benefit from immunotherapy and guide clinical decision-making.

## 7 Summary and prospects

Among patients with R/M CC, positive results are now available from two large phase III randomized controlled trials. The GOG-3016 study found that ICB monotherapy improved OS by 3.5 months compared to chemotherapy alone. The KEYNOTE-826 study showed that ICB in combination with chemotherapy with or without anti-angiogenic therapy improves OS by nearly 1 year compared to CCRT alone. However, the results of current studies are inconsistent, and more studies are needed to identify populations that would benefit from these treatments. Squamous cell carcinoma (SCC) is the main histologic subtype of CC, and previous studies have shown that SCC is most likely to express PD-L1 and is more sensitive to ICBs (106). Several studies have identified differences in treatment efficacy in patients with different levels of PD-L1 expression. This suggests that stratified studies based on CPS can be performed to explore the possibility of using ICB monotherapy to treat sensitive populations. Overall, while combination therapy may be more beneficial than monotherapy, it is important to be aware of the potential increase in drug-related toxicity. In addition, as a result of the large CC patient population in China, some new drugs have only been developed and tested in Chinese patients. Several trials have yielded good results, and international multi-center studies are expected to test these drugs in more diverse patient populations.

Concurrent radiotherapy is the standard treatment modality for LACC patients, however, the efficacy is limited, especially for those with stage III/IV disease (6). Thus, the addition of immunotherapy may be of particular benefit to late-stage patients. Among patients with lymph node metastases or local tumor infiltration, the combination of ICB and CCRT is shown to have a “1+1>2” effect and increase the local control rate to reduce recurrence. Meanwhile, stage IB3-IIA patients may not require immunotherapy because CCRT alone has a high local control rate. The timing of immunotherapy, including immune induction, concurrent, and maintenance therapy, also deserve further exploration. Current immunotherapy for LACC is in phase I clinical trials with a fair safety profile. More results are expected to answer these questions.

There are several issues to consider regarding CC-specific immunotherapy. First, neuroendocrine CC, a rare pathological type that differs from SCC, has a poor prognosis and no standard treatment modality (107). Immune combination therapies have shown some promise in small sample retrospective studies and case reports (108, 109), however, larger randomized controlled trials are required to verify the efficacy of these drugs. Second, while T-cell-based immunotherapy has been most commonly studied, recent reports have shown that B cell infiltration and the formation of tertiary lymphoid structures (TLS) in melanoma and soft tissue sarcoma were involved in tumor immune responses. Large-scale RNA sequencing has also shown that tumor-infiltrating B cells and TLS correlated positively with patient response to ICB therapy and prognosis (110–113). Thus, it may be worthwhile exploring potential immunotherapy options associated with other immune cells, including B cells, and their interaction with HPV. Third, new evidence suggests that the route of administration and drug dosage forms can be improved through the use of nanomaterials (114, 115). In addition, by enriching public databases and developing novel computer algorithms, several studies have established various models and systems for screening tumor-associated neoantigens and quantitatively evaluating TME (116, 117). One study used deep learning artificial intelligence-based algorithms to analyze the CheckMate-038 trial and found that it could predict the expression of tumor-specific T-cell receptors in melanoma patients receiving immunotherapy (118). Most of these new technologies are in the exploratory stage and may be used to test drug efficacy, predict adverse effects (119–121), screen for potential biomarkers, and aid in drug development.

In conclusion, immunotherapy has shown safety and some efficacy in patients with LACC and R/M CC. However, additional large multicenter clinical trials are needed to identify more first- and second-line treatment modalities and to screen for patient populations suitable for immunotherapy. This will be critical to prolonging the survival and improving the quality of life of CC patients.

## Author contributions

All authors contributed to the study conception and design. ZS and KZ performed the literature search and data analysis. ZS and KZ wrote the first draft of the manuscript. ZS created the original figure and tables. LZ critically revised the work. All authors contributed to the article and approved the submitted version.

## Supplementary material

SUPPLEMENTARY TABLE 1 Phase I/II clinical trial of immune checkpoint drugs to treat locally advanced cervical cancer (derived from clinicaltrials.gov, accessed 12 Sep 2022).

SUPPLEMENTARY TABLE 2 Phase I/II clinical trial of immune checkpoint drugs to treat recurrent/metastatic cervical cancer (derived from clinicaltrials.gov, accessed 12 Sep 2022).

SUPPLEMENTARY TABLE 3 A glossary of abbreviations.

## References

[1] Sung H Ferlay J Siegel RL Laversanne M Soerjomataram I Jemal A. Global cancer statistics 2020: GLOBOCAN estimates of incidence and mortality worldwide for 36 cancers in 185 countries. CA Cancer J Clin (2021) 71:209–49. doi: 10.3322/caac.21660 33538338

[2] Xia C Dong X Li H Cao M Sun D He S. Cancer statistics in China and united states, 2022: profiles, trends, and determinants. Chin Med J (Engl) (2022) 135:584–90. doi: 10.1097/CM9.0000000000002108 PMC8920425 35143424

[3] Zheng R Zhang S Zeng H Wang S Sun K Chen R. Cancer incidence and mortality in China, 2016. J Natl Cancer Center (2022) 2:1–9. doi: 10.1016/j.jncc.2022.02.002 PMC11256658 39035212

[4] Liu Y Wu L Tong R Yang F Yin L Li M. PD-1/PD-L1 inhibitors in cervical cancer. Front Pharmacol (2019) 10:65. doi: 10.3389/fphar.2019.00065 30774597 PMC6367228

[5] Ibrahim A Rask Pukkala E Aro AR. Predictors of cervical cancer being at an advanced stage at diagnosis in Sudan. Int J Womens Health (2011) 3:385. doi: 10.2147/IJWH.S21063 22140326 PMC3225468

[6] Pujade-Lauraine E Tan DS Leary A Mirza MR Enomoto T Takyar J. Comparison of global treatment guidelines for locally advanced cervical cancer to optimize best care practices: A systematic and scoping review. Gynecol Oncol (2022) S0090-8258(22):00552-2. doi: 10.1016/j.ygyno.2022.08.013 36096973

[7] Zhou Y Rassy E Coutte A Achkar S Espenel S Genestie C. Current standards in the management of early and locally advanced cervical cancer: Update on the benefit of Neoadjuvant/Adjuvant strategies. Cancers (Basel) (2022) 14:2449. doi: 10.3390/cancers14102449 35626051 PMC9139662

[8] Tian T Gong X Gao X Li Y Ju W Ai Y. Comparison of survival outcomes of locally advanced cervical cancer by histopathological types in the surveillance, epidemiology, and end results (SEER) database: a propensity score matching study. Infect Agent Cancer (2020) 15:33. doi: 10.1186/s13027-020-00299-3 32435273 PMC7222537

[9] Mayadev JS Ke G Mahantshetty U Pereira MD Tarnawski R Toita T. Global challenges of radiotherapy for the treatment of locally advanced cervical cancer. Int J Gynecol Cancer (2022) 32:436–45. doi: 10.1136/ijgc-2021-003001 PMC8921593 35256434

[10] Boussios S Seraj E Zarkavelis G Petrakis D Kollas A Kafantari A. Management of patients with recurrent/advanced cervical cancer beyond first line platinum regimens: Where do we stand? a literature review. Crit Rev Oncol/Hematol (2016) 108:164–74. doi: 10.1016/j.critrevonc.2016.11.006 27931835

[11] Gennigens C Jerusalem G Lapaille L de Cuypere M Streel S Kridelka F. Recurrent or primary metastatic cervical cancer: current and future treatments. ESMO Open (2022) 7:100579. doi: 10.1016/j.esmoop.2022.100579 36108558 PMC9588874

[12] da Costa SC Bonadio RC Gabrielli FC Aranha AS Dias Genta ML Miranda VC. Neoadjuvant chemotherapy with cisplatin and gemcitabine followed by chemoradiation versus chemoradiation for locally advanced cervical cancer: A randomized phase II trial. JCO (2019) 37:3124–31. doi: 10.1200/JCO.19.00674 31449470

[13] Mileshkin LR Moore KN Barnes E Gebski V Narayan K Bradshaw N. Adjuvant chemotherapy following chemoradiation as primary treatment for locally advanced cervical cancer compared to chemoradiation alone: The randomized phase III OUTBACK trial (ANZGOG 0902, RTOG 1174, NRG 0274). JCO (2021) 39:LBA3–3. doi: 10.1200/JCO.2021.39.15_suppl.LBA3

[14] Čerina D Boraska Jelavić T Buljubašić Franić M Tomić K Bajić Ž Vrdoljak E. Is there a place for adjuvant chemotherapy in the treatment of locally advanced cervical cancer? Curr Oncol (2022) 29:5223–37. doi: 10.3390/curroncol29080415 PMC9332289 35892984

[15] Lu H Wu Y Liu X Huang H Jiang H Zhu C. Endostar, an antiangiogenesis inhibitor, combined with chemoradiotherapy for locally advanced cervical cancer. Oncol Res (2022) 28:929–44. doi: 10.3727/096504021X16318716607908 PMC8790112 34544526

[16] Schefter T Winter K Kwon JS Stuhr K Balaraj K Yaremko BP. RTOG 0417: efficacy of bevacizumab in combination with definitive radiation therapy and cisplatin chemotherapy in untreated patients with locally advanced cervical carcinoma. Int J Radiat Oncol Biol Phys (2014) 88:101–5. doi: 10.1016/j.ijrobp.2013.10.022 24331655

[17] Seamon LG Java JJ Monk BJ Penson RT Brown J Mannel RS. Impact of tumour histology on survival in advanced cervical carcinoma: an NRG Oncology/Gynaecologic oncology group study. Br J Cancer (2018) 118:162–70. doi: 10.1038/bjc.2017.400 PMC5785748 29182608

[18] Tewari KS Sill MW Penson RT Huang H Ramondetta LM Landrum LM. Bevacizumab for advanced cervical cancer: final overall survival and adverse event analysis of a randomised, controlled, open-label, phase 3 trial (Gynecologic oncology group 240). Lancet (2017) 390:1654–63. doi: 10.1016/S0140-6736(17)31607-0 PMC5714293 28756902

[19] Colombo N Dubot C Lorusso D Caceres MV Hasegawa K Shapira-Frommer R. Pembrolizumab for persistent, recurrent, or metastatic cervical cancer. N Engl J Med (2021) 385:1856–67. doi: 10.1056/NEJMoa2112435 34534429

[20] Walboomers JM Jacobs MV Manos MM Bosch FX Kummer JA Shah KV. Human papillomavirus is a necessary cause of invasive cervical cancer worldwide. J Pathol (1999) 189:12–9. doi: 10.1002/(SICI)1096-9896(199909)189:1<12:AID-PATH431>3.0.CO;2-F 10451482

[21] Yuan Y Cai X Shen F Ma F. HPV post-infection microenvironment and cervical cancer. Cancer Lett (2021) 497:243–54. doi: 10.1016/j.canlet.2020.10.034 33122098

[22] Petitprez F Meylan M de Reyniès A Sautès-Fridman C Fridman WH. The tumor microenvironment in the response to immune checkpoint blockade therapies. Front Immunol (2020) 11:784. doi: 10.3389/fimmu.2020.00784 32457745 PMC7221158

[23] Tumeh PC Harview CL Yearley JH Shintaku IP Taylor EJ Robert L. PD-1 blockade induces responses by inhibiting adaptive immune resistance. Nature (2014) 515:568–71. doi: 10.1038/nature13954 PMC4246418 25428505

[24] Zhou J Lei N Tian W Guo R Chen M Qiu L. Recent progress of the tumor microenvironmental metabolism in cervical cancer radioresistance. Front Oncol (2022) 12:999643. doi: 10.3389/fonc.2022.999643 36313645 PMC9597614

[25] Shamseddine AA Burman B Lee NY Zamarin D Riaz N. Tumor immunity and immunotherapy for HPV-related cancers. Cancer Discovery (2021) 11:1896–912. doi: 10.1158/2159-8290.CD-20-1760 PMC8338882 33990345

[26] Pardoll DM. The blockade of immune checkpoints in cancer immunotherapy. Nat Rev Cancer (2012) 12:252–64. doi: 10.1038/nrc3239 PMC4856023 22437870

[27] Chen X Wu W Wei W Zou L. Immune checkpoint inhibitors in peripheral T-cell lymphoma. Front Pharmacol (2022) 13:869488. doi: 10.3389/fphar.2022.869488 35559250 PMC9086454

[28] Wei SC Duffy CR Allison JP. Fundamental mechanisms of immune checkpoint blockade therapy. Cancer Discovery (2018) 8:1069–86. doi: 10.1158/2159-8290.CD-18-0367 30115704

[29] Yuan L Tatineni J Mahoney KM Freeman GJ. VISTA: A mediator of quiescence and a promising target in cancer immunotherapy. Trends Immunol (2021) 42:209–27. doi: 10.1016/j.it.2020.12.008 PMC8088836 33495077

[30] Shibru B Fey K Fricke S Blaudszun A-R Fürst F Weise M. Detection of immune checkpoint receptors - a current challenge in clinical flow cytometry. Front Immunol (2021) 12:694055. doi: 10.3389/fimmu.2021.694055 34276685 PMC8281132

[31] Naimi A Mohammed RN Raji A Chupradit S Yumashev AV Suksatan W. Tumor immunotherapies by immune checkpoint inhibitors (ICIs); the pros and cons. Cell Commun Signal (2022) 20:44. doi: 10.1186/s12964-022-00854-y 35392976 PMC8991803

[32] Santin AD Deng W Frumovitz M Buza N Bellone S Huh W. Phase II evaluation of nivolumab in the treatment of persistent or recurrent cervical cancer (NCT02257528/NRG-GY002). Gynecol Oncol (2020) 157:161–6. doi: 10.1016/j.ygyno.2019.12.034 PMC7127981 31924334

[33] Chung HC Ros W Delord J-P Perets R Italiano A Shapira-Frommer R. Efficacy and safety of pembrolizumab in previously treated advanced cervical cancer: Results from the phase II KEYNOTE-158 study. J Clin Oncol Off J Am Soc Clin Oncol (2019) 37:1470–8. doi: 10.1200/JCO.18.01265 30943124

[34] Naumann RW Hollebecque A Meyer T Devlin M-J Oaknin A Kerger J. Safety and efficacy of nivolumab monotherapy in recurrent or metastatic cervical, vaginal, or vulvar carcinoma: Results from the phase I/II CheckMate 358 trial. J Clin Oncol Off J Am Soc Clin Oncol (2019) 37:2825–34. doi: 10.1200/JCO.19.00739 PMC6823884 31487218

[35] Tamura K Hasegawa K Katsumata N Matsumoto K Mukai H Takahashi S. Efficacy and safety of nivolumab in Japanese patients with uterine cervical cancer, uterine corpus cancer, or soft tissue sarcoma: Multicenter, open-label phase 2 trial. Cancer Sci (2019) 110:2894–904. doi: 10.1111/cas.14148 PMC6726684 31348579

[36] Chen X Xue L Ding X Zhang J Jiang L Liu S. An fc-competent anti-human TIGIT blocking antibody ociperlimab (BGB-A1217) elicits strong immune responses and potent anti-tumor efficacy in pre-clinical models. Front Immunol (2022) 13:828319. doi: 10.3389/fimmu.2022.828319 35273608 PMC8902820

[37] Diab A Hamid O Thompson JA Ros W Eskens FA Doi T. Open-label, dose-escalation study of the OX40 agonist ivuxolimab in patients with locally advanced or metastatic cancers. Clin Cancer Res (2021) 28:71–83. doi: 10.1158/1078-0432.CCR-21-0845. clincanres.0845.2021. PMC9401502 34615725

[38] Liu J Liu N Wang D Li D Fang C. Efficacy and safety of QL1706, a novel dual immune checkpoint blockade containing a mixture of anti-PD1 IgG4 and anti-CTLA4 IgG1 antibodies, for advanced cervical cancer: Cohort data from a phase 1b trial. JCO (2022) 40:5535. doi: 10.1200/JCO.2022.40.16_suppl.5535

[39] Choi MC Kim Y-M Lee J-W Lee YJ Suh DH Lee SJ. Real-world experience of pembrolizumab monotherapy in patients with recurrent or persistent cervical cancer: A Korean multi-center retrospective study (KGOG1041). Cancers (Basel) (2020) 12:3188. doi: 10.3390/cancers12113188 33138190 PMC7693862

[40] Tewari KS Monk BJ Vergote I Miller A de Melo AC Kim H-S. Survival with cemiplimab in recurrent cervical cancer. N Engl J Med (2022) 386:544–55. doi: 10.1056/NEJMoa2112187 35139273

[41] O'Malley DM Oaknin A Monk BJ Selle F Rojas C Gladieff L. Phase II study of the safety and efficacy of the anti-PD-1 antibody balstilimab in patients with recurrent and/or metastatic cervical cancer. Gynecol Oncol (2021) 163:274–80. doi: 10.1016/j.ygyno.2021.08.018 34452745

[42] Lheureux S Butler MO Clarke B Cristea MC Martin LP Tonkin K. Association of ipilimumab with safety and antitumor activity in women with metastatic or recurrent human papillomavirus-related cervical carcinoma. JAMA Oncol (2018) 4:e173776. doi: 10.1001/jamaoncol.2017.3776 29145543 PMC6145732

[43] An J Guiling L Tang J Li BX Xiong HH Qiu H. Efficacy and safety of the anti–PD-L1 monoclonal antibody socazolimab for recurrent or metastatic cervical cancer: Results from the phase I dose-escalation and expansion study. J Clin Oncol Off J Am Soc Clin Oncol (2022) 40:5526. doi: 10.1200/JCO.2022.40.16_suppl.5526 36136294

[44] An J Tang J Li BX Xiong H Qiu H Luo L. Efficacy and safety of the anti-PD-L1 monoclonal antibody socazolimab for recurrent or metastatic cervical cancer: A phase I dose-escalation and expansion study. Clin Cancer Res (2022) 28:5098–106. doi: 10.1158/1078-0432.CCR-22-1280 36136294

[45] Wu X Ji J Lou H Li Y Feng M Xu N. Efficacy and safety of cadonilimab, an anti-PD-1/CTLA4 bi-specific antibody, in previously treated recurrent or metastatic (R/M) cervical cancer: a multicenter, open-label, single-arm, phase II trial (075). Gynecol Oncol (2022) 166:S47–8. doi: 10.1016/S0090-8258(22)01293-8

[46] Strauss J Gatti-Mays ME Cho BC Hill A Salas S McClay E. Bintrafusp alfa, a bifunctional fusion protein targeting TGF-β and PD-L1, in patients with human papillomavirus-associated malignancies. J Immunother Cancer (2020) 8:e001395. doi: 10.1136/jitc-2020-001395 33323462 PMC7745517

[47] National Cancer Institute. Phase II trial of M7824 in subjects with HPV associated malignancies: NCT03427411, 180056 (2022). Available at: https://clinicaltrials.gov/ct2/show/NCT03427411.

[48] Rischin D Gil-Martin M González-Martin A Braña I Hou JY Cho D. PD-1 blockade in recurrent or metastatic cervical cancer: Data from cemiplimab phase I expansion cohorts and characterization of PD-L1 expression in cervical cancer. Gynecol Oncol (2020) 159:322–8. doi: 10.1016/j.ygyno.2020.08.026 32917410

[49] Tewari KS Monk BJ Vergote I Miller A de Melo AC Kim HS. VP4-2021: EMPOWER-cervical 1/GOG-3016/ENGOT-cx9: Interim analysis of phase III trial of cemiplimab vs. investigator's choice (IC) chemotherapy (chemo) in recurrent/metastatic (R/M) cervical carcinoma. Ann Oncol (2021) 32:940–1. doi: 10.1016/j.annonc.2021.04.009

[50] Keam SJ. Cadonilimab: First approval. Drugs (2022) 82:1333–9. doi: 10.1007/s40265-022-01761-9 35986837

[51] Lin C-C Doi T Muro K Hou M-M Esaki T Hara H. Bintrafusp Alfa, a bifunctional fusion protein targeting TGFβ and PD-L1, in patients with esophageal squamous cell carcinoma: Results from a phase 1 cohort in Asia. Target Oncol (2021) 16:447–59. doi: 10.1007/s11523-021-00810-9 PMC8266718 33840050

[52] Strauss J Heery CR Schlom J Madan RA Cao L Kang Z. Phase I trial of M7824 (MSB0011359C), a bifunctional fusion protein targeting PD-L1 and TGFβ, in advanced solid tumors. Clin Cancer Res (2018) 24:1287–95. doi: 10.1158/1078-0432.CCR-17-2653 PMC7985967 29298798

[53] Wu L Wang P-H Hsiao S-Y Chang C-L Kim H-S Lee J-Y. AdvanTIG-202: A phase 2 study investigating anti-T cell immunoglobulin and ITIM domain monoclonal antibody ociperlimab plus tislelizumab in patients with previously treated recurrent or metastatic cervical cancer (333). Gynecol Oncol (2022) 166:S172. doi: 10.1016/S0090-8258(22)01555-4 40411966

[54] Zhang Z Liu X Chen D Yu J. Radiotherapy combined with immunotherapy: the dawn of cancer treatment. Signal Transduct Target Ther (2022) 7:258. doi: 10.1038/s41392-022-01102-y 35906199 PMC9338328

[55] Merlano MC Denaro N Galizia D Ruatta F Occelli M Minei S. How chemotherapy affects the tumor immune microenvironment: A narrative review. Biomedicines (2022) 10:1822. doi: 10.3390/biomedicines10081822 36009369 PMC9405073

[56] Huang Y Kim BY Chan CK Hahn SM Weissman IL Jiang W. Improving immune-vascular crosstalk for cancer immunotherapy. Nat Rev Immunol (2018) 18:195–203. doi: 10.1038/nri.2017.145 29332937 PMC5922422

[57] Tewari KS Colombo N Monk BJ Dubot C Cáceres MV Hasegawa K. Pembrolizumab + chemotherapy in patients with persistent, recurrent, or metastatic cervical cancer: Subgroup analysis of KEYNOTE-826. J Clin Oncol Off J Am Soc Clin Oncol (2022) 40:5506. doi: 10.1200/JCO.2022.40.16_suppl.5506

[58] Wang J Lou H Cai H-B Huang X Li G Wang L. A study of AK104 (an anti-PD1 and anti-CTLA4 bispecific antibody) combined with standard therapy for the first-line treatment of persistent, recurrent, or metastatic cervical cancer (R/M CC). J Clin Oncol Off J Am Soc Clin Oncol (2022) 40:106. doi: 10.1200/JCO.2022.40.16_suppl.106

[59] Lan C Shen J Wang Y Li J Liu Z He M. Camrelizumab plus apatinib in patients with advanced cervical cancer (CLAP): A multicenter, open-label, single-arm, phase II trial. J Clin Oncol (2020) 38:4095–106. doi: 10.1200/JCO.20.01920 PMC7768345 33052760

[60] Xu Q Wang J Sun Y Lin Y Liu J Zhuo Y. Efficacy and safety of sintilimab plus anlotinib for PD-L1-Positive recurrent or metastatic cervical cancer: A multicenter, single-arm, prospective phase II trial. J Clin Oncol Off J Am Soc Clin Oncol (2022) 40:1795–805. doi: 10.1200/JCO.21.02091 PMC9148684 35192397

[61] Cheng M Wang H Zhao Y Li G. Efficacy and prognostic factors for response to PD-1 inhibitors in advanced cervical carcinoma: A retrospective study. Drug Des Devel Ther (2022) 16:887–97. doi: 10.2147/DDDT.S358302 PMC8976502 35378925

[62] Oaknin A Backes F van Nieuwenhuysen E Eskander R González-Martín A Makker V. LIO-1: Initial phase 2 experience of lucitanib + nivolumab in patients with metastatic or recurrent cervical cancer (NCT04042116; ENGOT-GYN3/AGO/LIO) (034). Gynecol Oncol (2022) 166:S24. doi: 10.1016/S0090-8258(22)01252-5

[63] An J Wu L Li X Wang J Hou X Wang Q. Efficacy and safety of serplulimab (an anti-PD-1 antibody) combined with albumin-bound paclitaxel in patients with advanced cervical cancer who have progressive disease or intolerable toxicity after first-line standard chemotherapy (074). Gynecol Oncol (2022) 166:S47. doi: 10.1016/S0090-8258(22)01292-6

[64] Friedman CF Snyder Charen A Zhou Q Carducci MA Buckley De Meritens A Corr BR. Phase II study of atezolizumab in combination with bevacizumab in patients with advanced cervical cancer. J Immunother Cancer (2020) 8:e001126. doi: 10.1136/jitc-2020-001126 33004542 PMC7534695

[65] de Jaeghere EA Tuyaerts S van Nuffel AM Belmans A Bogaerts K Baiden-Amissah R. Pembrolizumab, radiotherapy, and an immunomodulatory five-drug cocktail in pretreated patients with persistent, recurrent, or metastatic cervical or endometrial carcinoma: Results of the phase II PRIMMO study. Cancer Immunol Immunother (2022). doi: 10.1007/s00262-022-03253-x PMC9870976 35960332

[66] O'Malley DM Neffa M Monk BJ Melkadze T Huang M Kryzhanivska A. Dual PD-1 and CTLA-4 checkpoint blockade using balstilimab and zalifrelimab combination as second-line treatment for advanced cervical cancer: An open-label phase II study. J Clin Oncol Off J Am Soc Clin Oncol (2022) 40:762–71. doi: 10.1200/JCO.21.02067 PMC8887945 34932394

[67] Lorusso D Vergote I O'Cearbhaill RE Westermann AM Banerjee SN van Nieuwenhuysen E. Tisotumab vedotin (TV) + pembrolizumab (pembro) in first-line (1L) recurrent or metastatic cervical cancer (r/mCC): Interim results of ENGOT Cx8/GOG 3024/innovaTV 205. J Clin Oncol Off J Am Soc Clin Oncol (2022) 40:5507. doi: 10.1200/JCO.2022.40.16_suppl.5507

[68] Youn JW Hur S-Y Woo JW Kim Y-M Lim MC Park SY. Pembrolizumab plus GX-188E therapeutic DNA vaccine in patients with HPV-16-positive or HPV-18-positive advanced cervical cancer: interim results of a single-arm, phase 2 trial. Lancet Oncol (2020) 21:1653–60. doi: 10.1016/S1470-2045(20)30486-1 33271094

[69] Park JS Hur S-Y Lim MC Kim YM No JH Kim B-G. Efficacy and safety results of GX-188E, a therapeutic DNA vaccine, combined with pembrolizumab administration in patients with HPV 16- and/or 18- positive advanced cervical cancer: Phase II interim analysis results (KEYNOTE-567). JCO (2021) 39:5511. doi: 10.1200/JCO.2021.39.15_suppl.5511

[70] Yin H Guo W Sun X Li R Feng C Tan Y. TILs and anti-PD1 therapy: An alternative combination therapy for PDL1 negative metastatic cervical cancer. J Immunol Res (2020) 2020:8345235. doi: 10.1155/2020/8345235 32964058 PMC7492938

[71] National Comprehensive Cancer Network. NCCN clinical practice guidelines in oncology: Cervical cancer version 1.2022 (2022). Available at: https://www.nccn.org/guidelines/guidelines-detail?category=1&id=1426. 10.6004/jnccn.2022.0020 35390769

[72] Zhang L Mai W Jiang W Geng Q. Sintilimab: A promising anti-tumor PD-1 antibody. Front Oncol (2020) 10:594558. doi: 10.3389/fonc.2020.594558 33324564 PMC7726413

[73] Zhu J Yang Q Xu W. Iterative upgrading of small molecular tyrosine kinase inhibitors for EGFR mutation in NSCLC: Necessity and perspective. Pharmaceutics (2021) 13:1500. doi: 10.3390/pharmaceutics13091500 34575576 PMC8468657

[74] Grau JF Farinas-Madrid L Oaknin A. A randomized phase III trial of platinum chemotherapy plus paclitaxel with bevacizumab and atezolizumab versus platinum chemotherapy plus paclitaxel and bevacizumab in metastatic (stage IVB), persistent, or recurrent carcinoma of the cervix: the BEATcc study (ENGOT-Cx10/GEICO 68-C/JGOG1084/GOG-3030). Int J Gynecol Cancer (2020) 30:139–43. doi: 10.1136/ijgc-2019-000880 31645423

[75] Tuyaerts S van Nuffel AM Naert E van Dam PA Vuylsteke P de Caluwé A. PRIMMO study protocol: a phase II study combining PD-1 blockade, radiation and immunomodulation to tackle cervical and uterine cancer. BMC Cancer (2019) 19:506. doi: 10.1186/s12885-019-5676-3 31138229 PMC6537207

[76] Kaplon H Muralidharan M Schneider Z Reichert JM. Antibodies to watch in 2020. MAbs (2020) 12:1703531. doi: 10.1080/19420862.2019.1703531 31847708 PMC6973335

[77] O'Malley DM Oaknin A Monk BJ Leary A Selle F Alexandre J. Single-agent anti-PD-1 balstilimab or in combination with anti-CTLA-4 zalifrelimab for recurrent/metastatic (R/M) cervical cancer (CC): Preliminary results of two independent phase II trials. Ann Oncol (2020) 31:S1164–5. doi: 10.1016/j.annonc.2020.08.2264

[78] O'Malley DM Randall LM Jackson CG Coleman RL Hays JL Moore KN. RaPiDS (GOG-3028): randomized phase II study of balstilimab alone or in combination with zalifrelimab in cervical cancer. Future Oncol (London England) (2021) 17:3433–43. doi: 10.2217/fon-2021-0529 34409858

[79] Jung KH LoRusso P Burris H Gordon M Bang Y-J Hellmann MD. Phase I study of the indoleamine 2,3-dioxygenase 1 (IDO1) inhibitor navoximod (GDC-0919) administered with PD-L1 inhibitor (Atezolizumab) in advanced solid tumors. Clin Cancer Res (2019) 25:3220–8. doi: 10.1158/1078-0432.CCR-18-2740 PMC7980952 30770348

[80] Coleman RL Lorusso D Gennigens C González-Martín A Randall L Cibula D. Efficacy and safety of tisotumab vedotin in previously treated recurrent or metastatic cervical cancer (innovaTV 204/GOG-3023/ENGOT-cx6): a multicentre, open-label, single-arm, phase 2 study. Lancet Oncol (2021) 22:609–19. doi: 10.1016/S1470-2045(21)00056-5 33845034

[81] Vergote IB Concin N Mirza MR Malmberg A Eaton L Nicacio L. Phase I/II trial of tisotumab vedotin plus bevacizumab, pembrolizumab, or carboplatin in recurrent or metastatic cervical cancer (innovaTV 205/ENGOT-cx8). Ann Oncol (2019) 30:v433–4. doi: 10.1093/annonc/mdz250.072

[82] Shanmugasundaram S You J. Targeting persistent human papillomavirus infection. Viruses (2017) 9:229. doi: 10.3390/v9080229 28820433 PMC5580486

[83] Morgensztern D Herbst RS. Nivolumab and pembrolizumab for non-small cell lung cancer. Clin Cancer Res (2016) 22:3713–7. doi: 10.1158/1078-0432.CCR-15-2998 27252413

[84] Mayadev J Nunes AT Li M Marcovitz M Lanasa MC Monk BJ. CALLA: Efficacy and safety of concurrent and adjuvant durvalumab with chemoradiotherapy versus chemoradiotherapy alone in women with locally advanced cervical cancer: a phase III, randomized, double-blind, multicenter study. Int J Gynecol Cancer (2020) 30:1065–70. doi: 10.1136/ijgc-2019-001135 PMC7398223 32447296

[85] Okonogi N Usui H Murata K Hori M Kurokawa T Fujiwara T. Phase ib study of durvalumab (MEDI4736) in combination with carbon-ion radiotherapy and weekly cisplatin for patients with locally advanced cervical cancer (DECISION study): study protocol for a prospective open-label single-arm study. BMJ Open (2022) 12:e056424. doi: 10.1136/bmjopen-2021-056424 PMC8896055 35236732

[86] Duska LR Scalici JM Temkin SM Schwarz JK Crane EK Moxley KM. Results of an early safety analysis of a study of the combination of pembrolizumab and pelvic chemoradiation in locally advanced cervical cancer. Cancer (2020) 126:4948–56. doi: 10.1002/cncr.33136 32910478

[87] Rodrigues M Loap P Dubot C Durdux C Bazire L Minsat M. Combination of nivolumab with chemoradiotherapy for locally advanced cervical cancer: NiCOL phase I trial. JCO (2022) 40:5534. doi: 10.1200/JCO.2022.40.16_suppl.5534 PMC10287640 37349318

[88] Yabuno A Nakamura K Satoh T Fujiwara H Kurosaki A Yamashita S. GOTIC-018: Phase I, open-label, multicenter study to assess the safety of pre- and co-administration of ONO-4538 (nivolumab) with concurrent chemoradiation (CCRT) in patients (pts) with locally advanced cervical carcinoma (LACvCa). J Clin Oncol Off J Am Soc Clin Oncol (2022) 40:5529. doi: 10.1200/JCO.2022.40.16_suppl.5529

[89] Ou D Xu H. Toripalimab combined with chemoradiotherapy for patients with locally advanced cervical squamous cell carcinoma. JCO (2022) 40:5538. doi: 10.1200/JCO.2022.40.16_suppl.5538

[90] Mayadev JS Enserro D Lin YG Da Silva DM Lankes HA Aghajanian C. Sequential ipilimumab after chemoradiotherapy in curative-intent treatment of patients with node-positive cervical cancer. JAMA Oncol (2019) 6:92–9. doi: 10.1001/jamaoncol.2019.3857 PMC6902184 31774464

[91] Mayadev J Zamarin D Deng W Lankes H Pesci G Park K. Safety and immunogenicity of anti PD-L1 (Atezolizumab) given as an immune primer or concurrently with extended field chemoradiotherapy for node positive locally advanced cervical cancer: an NRG oncology trial (024). Gynecol Oncol (2022) 166:S18–9. doi: 10.1016/S0090-8258(22)01242-2

[92] Chen J Li C Cao Y Zhu L Zhang B You J. Toripalimab combined with concurrent platinum-based chemoradiotherapy in patients with locally advanced cervical cancer: an open-label, single-arm, phase II trial. BMC Cancer (2022) 22:793. doi: 10.1186/s12885-022-09866-w 35854236 PMC9295395

[93] Da Silva DM Enserro DM Mayadev JS Skeate JG Matsuo K Pham HQ. Immune activation in patients with locally advanced cervical cancer treated with ipilimumab following definitive chemoradiation (GOG-9929). Clin Cancer Res (2020) 26:5621–30. doi: 10.1158/1078-0432.CCR-20-0776 PMC7642021 32816895

[94] Mayadev J Zamarin D Deng W Lankes H O'Cearbhaill R Aghajanian CA. Anti-PD-L1 (atezolizumab) as an immune primer and concurrently with extended-field chemoradiotherapy for node-positive locally advanced cervical cancer. Int J Gynecol Cancer (2020) 30:701–4. doi: 10.1136/ijgc-2019-001012 PMC7310564 31871115

[95] Davis AA Patel VG. The role of PD-L1 expression as a predictive biomarker: an analysis of all US food and drug administration (FDA) approvals of immune checkpoint inhibitors. J Immunother Cancer (2019) 7:278. doi: 10.1186/s40425-019-0768-9 31655605 PMC6815032

[96] Wang R Zhang Y Shan F. PD-L1: Can it be a biomarker for the prognosis or a promising therapeutic target in cervical cancer? Int Immunopharmacol (2022) 103:108484. doi: 10.1016/j.intimp.2021.108484 34954558

[97] Goodman AM Kato S Bazhenova L Patel SP Frampton GM Miller V. Tumor mutational burden as an independent predictor of response to immunotherapy in diverse cancers. Mol Cancer Ther (2017) 16:2598–608. doi: 10.1158/1535-7163.MCT-17-0386 PMC5670009 28835386

[98] Chan TA Yarchoan M Jaffee E Swanton C Quezada SA Stenzinger A. Development of tumor mutation burden as an immunotherapy biomarker: utility for the oncology clinic. Ann Oncol (2019) 30:44–56. doi: 10.1093/annonc/mdy495 30395155 PMC6336005

[99] Huang X He M Peng H Tong C Liu Z Zhang X. Genomic profiling of advanced cervical cancer to predict response to programmed death-1 inhibitor combination therapy: a secondary analysis of the CLAP trial. J Immunother Cancer (2021) 9:e002223. doi: 10.1136/jitc-2020-002223 34011535 PMC8137235

[100] Walk EE Yohe SL Beckman A Schade A Zutter MM Pfeifer J. The cancer immunotherapy biomarker testing landscape. Arch Pathol Lab Med (2020) 144:706–24. doi: 10.5858/arpa.2018-0584-CP 31714809

[101] Otter SJ Chatterjee J Stewart AJ Michael A. The role of biomarkers for the prediction of response to checkpoint immunotherapy and the rationale for the use of checkpoint immunotherapy in cervical cancer. Clin Oncol (R Coll Radiol) (2019) 31:834–43. doi: 10.1016/j.clon.2019.07.003 31331818

[102] Dudley JC Lin M-T Le DT Eshleman JR. Microsatellite instability as a biomarker for PD-1 blockade. Clin Cancer Res (2016) 22:813–20. doi: 10.1158/1078-0432.CCR-15-1678 26880610

[103] Howitt BE Sun HH Roemer MG Kelley A Chapuy B Aviki E. Genetic basis for PD-L1 expression in squamous cell carcinomas of the cervix and vulva. JAMA Oncol (2016) 2:518–22. doi: 10.1001/jamaoncol.2015.6326 26913631

[104] Wang Y-M Qiu J-J Qu X-Y Peng J Lu C Zhang M. Accumulation of dysfunctional tumor-infiltrating PD-1+ DCs links PD-1/PD-L1 blockade immunotherapeutic response in cervical cancer. Oncoimmunology (2022) 11:2034257. doi: 10.1080/2162402X.2022.2034257 35154907 PMC8837238

[105] Cristescu R Mogg R Ayers M Albright A Murphy E Yearley J. Pan-tumor genomic biomarkers for PD-1 checkpoint blockade-based immunotherapy. Science (2018) 362:eaar3593. doi: 10.1126/science.aar3593 30309915 PMC6718162

[106] Kim M Kim H Suh DH Kim K Kim H Kim YB. Identifying rational candidates for immunotherapy targeting PD-1/PD-L1 in cervical cancer. Anticancer Res (2017) 37:5087–94. doi: 10.21873/anticanres.11926 28870938

[107] Tempfer CB Tischoff I Dogan A Hilal Z Schultheis B Kern P. Neuroendocrine carcinoma of the cervix: a systematic review of the literature. BMC Cancer (2018) 18:530. doi: 10.1186/s12885-018-4447-x 29728073 PMC5935948

[108] Paterniti TA Dorr K Ullah A White J Williams H Ghamande S. Complete response to combination nivolumab and ipilimumab in recurrent neuroendocrine carcinoma of the cervix. Obstet Gynecol (2021) 138:813–6. doi: 10.1097/AOG.0000000000004573 34619736

[109] Al-Toubah T Halfdanarson T Gile J Morse B Sommerer K Strosberg J. Efficacy of ipilimumab and nivolumab in patients with high-grade neuroendocrine neoplasms. ESMO Open (2022) 7:100364. doi: 10.1016/j.esmoop.2021.100364 34973511 PMC8728436

[110] Petitprez F de Reyniès A Keung EZ Chen TW-W Sun C-M Calderaro J. B cells are associated with survival and immunotherapy response in sarcoma. Nature (2020) 577:556–60. doi: 10.1038/s41586-019-1906-8 31942077

[111] Helmink BA Reddy SM Gao J Zhang S Basar R Thakur R. B cells and tertiary lymphoid structures promote immunotherapy response. Nature (2020) 577:549–55. doi: 10.1038/s41586-019-1922-8 PMC8762581 31942075

[112] Cabrita R Lauss M Sanna A Donia M Skaarup Larsen M Mitra S. Tertiary lymphoid structures improve immunotherapy and survival in melanoma. Nature (2020) 577:561–5. doi: 10.1038/s41586-019-1914-8 31942071

[113] Vaghjiani RG Skitzki JJ. Tertiary lymphoid structures as mediators of immunotherapy response. Cancers (Basel) (2022) 14:3748. doi: 10.3390/cancers14153748 35954412 PMC9367241

[114] Wang N Wang Z Xu Z Chen X Zhu G. A cisplatin-loaded immunochemotherapeutic nanohybrid bearing immune checkpoint inhibitors for enhanced cervical cancer therapy. Angew Chem Int Ed Engl (2018) 57:3426–30. doi: 10.1002/anie.201800422 29405579

[115] Chen Z Yue Z Wang R Yang K Li S. Nanomaterials: A powerful tool for tumor immunotherapy. Front Immunol (2022) 13:979469. doi: 10.3389/fimmu.2022.979469 36072591 PMC9441741

[116] Roudko V Greenbaum B Bhardwaj N. Computational prediction and validation of tumor-associated neoantigens. Front Immunol (2020) 11:27. doi: 10.3389/fimmu.2020.00027 32117226 PMC7025577

[117] Kang Y Huang J Liu Y Zhang N Cheng Q Zhang Y. Integrated analysis of immune infiltration features for cervical carcinoma and their associated immunotherapeutic responses. Front Cell Dev Biol (2021) 9:573497. doi: 10.3389/fcell.2021.573497 33898414 PMC8063060

[118] Sidhom J-W Oliveira G Ross-MacDonald P Wind-Rotolo M Wu CJ Pardoll DM. Deep learning reveals predictive sequence concepts within immune repertoires to immunotherapy. Sci Adv (2022) 8:eabq5089. doi: 10.1126/sciadv.abq5089 36112691 PMC9481116

[119] Heilbroner SP Few R Mueller J Chalwa J Charest F Suryadevara S. Predicting cardiac adverse events in patients receiving immune checkpoint inhibitors: a machine learning approach. J Immunother Cancer (2021) 9:e002545. doi: 10.1136/jitc-2021-002545 34607896 PMC8491414

[120] Mu W Jiang L Shi Y Tunali I Gray JE Katsoulakis E. Non-invasive measurement of PD-L1 status and prediction of immunotherapy response using deep learning of PET/CT images. J Immunother Cancer (2021) 9:e002118. doi: 10.1136/jitc-2020-002118 34135101 PMC8211060

[121] Kim N Lee ES Won SE Yang M Lee AJ Shin Y. Evolution of radiological treatment response assessments for cancer immunotherapy: From iRECIST to radiomics and artificial intelligence. Korean J Radiol (2022) 23:1089–101. doi: 10.3348/kjr.2022.0225 PMC9614294 36098343

