# Supplementary material for: Immune checkpoint blockade for locally advanced or recurrent/metastatic cervical cancer: An update on clinical data
Source: Front Oncol. 2022 Dec 22;12:1045481. doi: 10.3389/fonc.2022.1045481 (PMC9832370; doi:10.3389/fonc.2022.1045481)
Supplement: SUPPLEMENTARY TABLE 1 — Phase I/II clinical trial of immune checkpoint drugs to treat locally advanced cervical cancer (derived from clinicaltrials.gov, accessed 12 Sep 2022). [file Table_1.pdf]

Supplementary Table 1. Phase I/II clinical trial of immune checkpoint drugs in locally advanced cervical cancer (Derived from clinicaltrials.gov, accessed 12 Sep 2022).

NCT Number	Interventions	Targets	Enrollment	Stage	Status
NCT04254107	SEA-TGT + Sasanlimab	Anti-TIGIT + Anti-PD-1	397	Phase 1	Recruiting
NCT03104699	AGEN2034 (Balstilimab)	Anti-PD-1	211	Phase 1|Phase 2	Active, not recruiting
NCT03894215	AGEN2034 (Balstilimab) + AGEN1884 (Zalifrelimab)	Anti-PD-1 + Anti-CTLA-4	200	Phase 2	Recruiting
NCT03612791	Atezolizumab + Radiotherapy + Cisplatin	Anti-PD-L1	189	Phase 2	Recruiting
NCT04913337	NGM707 + Pembrolizumab	Anti-ILT2/ILT4 + Anti-PD-1	179	Phase 1|Phase 2	Recruiting
NCT02315066	PF-04518600 ± PF-05082566	OX40 (CD134) agonist + 4-1BB (CD137) agonist	174	Phase 1	Completed
NCT03495882	AGEN1884 (Zalifrelimab) + AGEN2034 (Balstilimab)	Anti-CTLA-4 + Anti-PD-1	154	Phase 1|Phase 2	Active, not recruiting
NCT03518606	Durvalumab + Tremelimumab + metronomic Vinorelbine	Anti-PD-L1 + Anti-CTLA-4	150	Phase 1|Phase 2	Active, not recruiting
NCT03833479	TSR-042(Dostarlimab-gxly)	Anti-PD-1	132	Phase 2	Recruiting
NCT05511623	Tislelizumab	Anti-PD-1	112	Phase 2	Not yet recruiting
NCT05492123	Nivolumab + Ipilimumab; Nivolumab + Chemoradiation	Anti-PD-1 + Anti-CTLA-4	112	Phase 2	Recruiting
NCT04516616	Neoadjuvant Camrelizumab (SHR-1210) + Cisplatin + Albumin-bound paclitaxel	Anti-PD-1	84	Phase 2	Recruiting
NCT04432597	PRGN-2009 + M7824 (Bintrafusp alfa)	HPV Vaccine + Anti-PD-L1 and TGF-β	76	Phase 1|Phase 2	Recruiting
NCT02291055	Axalimogene Filolisbac (ADXS11-001) + Durvalumab (MEDI4736)	HPV-E7 + Anti-PD-L1	66	Phase 1|Phase 2	Unknown
NCT04287868	PDS0101 + M7824 (Bintrafusp alfa) + NHS-IL12	HPV 16 + Anti-PD-L1 and TGF-β + IL12	56	Phase 1|Phase 2	Recruiting
NCT05504642	Pre-, Concurrent-Chemo-radio-immunotherapy, and Maintenance Treatment (Nivolumab/Ipilimumab)	Anti-PD-1/Anti-CTLA-4	55	Phase 2	Not yet recruiting
NCT04799639	Sindilimab + Paclitaxel + Cisplantin	Anti-PD-1	47	Phase 2	Not yet recruiting
NCT03946358	Atezolizumab + UCPVax	Anti-PD-L1 + Universal Cancer Peptides Vaccine	47	Phase 2	Recruiting
NCT04238988	Neoadjuvant Pembrolizumab + Carboplatin + Taxol (Paclitaxel)	Anti-PD-1	45	Phase 2	Recruiting
NCT03738228	Atezolizumab + Brachytherapy + Cisplatin + Radiation Therapy	Anti-PD-L1	40	Phase 1	Active, not recruiting
NCT04865887	Pembrolizumab + Lenvatinib	Anti-PD-1 + Anti-VEGFR	35	Phase 2	Recruiting
NCT01711515	Cisplatin + EBRT + Internal Radiation Therapy + Ipilimumab	Anti-CTLA-4	34	Phase 1	Completed
NCT04551950	M7824 (Bintrafusp alfa) + Carboplatin + Paclitaxel ± Bevacizumab | M7824 + Cisplatin + Radiotherapy	Anti-PD-L1 and TGF-β	25	Phase 1	Active, not recruiting
NCT03298893	Nivolumab + Cisplatin + radiotherapy	Anti-PD-1	21	Phase 1|Phase 2	Completed
NCT05105672	Sintilimab + Cisplatin + EBRT + Brachytherapy	Anti-PD-1	20	Phase 2	Recruiting
NCT05437692	Zimberelimab (GLS-010) + CCRT	Anti-PD-1	19	Phase 2	Recruiting

Abbreviations: EBRT, external beam radiotherapy; HPV, the human papillomavirus; PD-1, programmed cell death protein 1; PD-L1, programmed cell death ligand-1; CTLA-4, cytotoxic T-lymphocyte-associated protein 4; TIGIT, T cell immunoglobulin and ITIM domains; VEGFR, vascular endothelial growth factor receptor; CCRT, concurrent radiotherapy and chemotherapy; IL12, Interleukin 12; ILT, immunoglobulin-like transcript.
