# Supplementary material for: Immune checkpoint blockade for locally advanced or recurrent/metastatic cervical cancer: An update on clinical data
Source: Front Oncol. 2022 Dec 22;12:1045481. doi: 10.3389/fonc.2022.1045481 (PMC9832370; doi:10.3389/fonc.2022.1045481)
Supplement: SUPPLEMENTARY TABLE 2 — Phase I/II clinical trial of immune checkpoint drugs to treat recurrent/metastatic cervical cancer (derived from clinicaltrials.gov, accessed 12 Sep 2022). [file Table_2.pdf]

Supplementary Table 2. Phase I/II clinical trial of immune checkpoint drugs in recurrent/metastatic cervical cancer (Derived from clinicaltrials.gov, accessed 12 Sep 2022).

NCT Number	Interventions	Targets	Enrollment	Stage	Status
NCT02628067	Pembrolizumab	Anti-PD-1	1609	Phase 2	Recruiting
NCT05007106	Pembrolizumab/Vibostolimab Co-Formulation	Anti-PD-1 and TIGIT	610	Phase 2	Recruiting
NCT03829501	KY1044 + Atezolizumab	Anti-ICOS + Anti-PD-L1	412	Phase 1|Phase 2	Recruiting
NCT03219268	Tebotelimab	Anti-PD-1 and LAG-3	353	Phase 1	Active, not recruiting
NCT03827837	SHR-1210 (Camrelizumab) + Famitinib	Anti-PD-1 + Anti-VEGFR	265	Phase 2	Recruiting
NCT03849469	XmAb22841 + Pembrolizumab	Anti-LAG-3 and CTLA-4 + Anti-PD-1	242	Phase 1	Active, not recruiting
NCT04652076	NP137 + Pembrolizumab + Paclitaxel + Carboplatin	Anti-Netrin 1 + Anti-PD-1	240	Phase 1|Phase 2	Recruiting
NCT04042116	Lucitanib + Nivolumab	Anti-VEGFR and FGFR + Anti-PD-1	227	Phase 1|Phase 2	Active, not recruiting
NCT03104699	AGEN2034 (Balstilimab)	Anti-PD-1	211	Phase 1|Phase 2	Active, not recruiting
NCT03108495	LN-145 + pembrolizumab	TIL + Anti-PD-1	189	Phase 2	Recruiting
NCT04895709	BMS-986340 + BMS-936558-01 (Nivolumab)	Anti-CCR8 + Anti-PD-1	185	Phase 1|Phase 2	Recruiting
NCT05033132	Balstilimab + Zalifrelimab	Anti-PD-1 + Anti-CTLA-4	177	Phase 2	Recruiting
NCT04300647	Tiragolumab + Atezolizumab	Anti-TIGIT + Anti-PD-L1	172	Phase 2	Active, not recruiting
NCT04974944	Camrelizumab (SHR1210) + Apatinib	Anti-PD-1 + VEGFR-2	172	Phase 2	Recruiting
NCT04693234	Tislelizumab + Ociperlimab (BGB-A1217)	Anti-PD-1 + Anti-TIGIT	167	Phase 2	Active, not recruiting
NCT05383482	Afuresertib + Nab-paclitaxel/Docetaxel + Sintilimab	AKT Kinase Inhibitor + Anti-PD-1	167	Phase 1|Phase 2	Recruiting
NCT04772989	AB308 + Zimberelimab (AB122)	Anti-TIGIT + Anti-CTLA-4	160	Phase 1	Recruiting
NCT04680988	SHR-1210 (Camrelizumab) + SHR-1020 (Famitinib)	Anti-PD-1 + Anti-VEGFR	153	Phase 2	Recruiting
NCT05032040	XmAb20717	Anti-PD-1 and CTLA-4	150	Phase 2	Recruiting
NCT05337735	XmAb20717	Anti-PD-1 and CTLA-4	140	Phase 2	Recruiting
NCT05290935	Camrelizumab + Albumin-bound paclitaxel	Anti-PD-1	122	Phase 2	Recruiting
NCT05438420	Q702 + Pembrolizumab	Axl/Mer/CSF1R Selective TKI + Anti-PD-1	120	Phase 1|Phase 2	Not yet recruiting
NCT04357873	Pembrolizumab + Vorinostat	Anti-PD-1 + Anti-HDAC	112	Phase 2	Active, not recruiting
NCT04646005	Cemiplimab + ISA101b	Anti-PD-1 + HPV 16	105	Phase 2	Recruiting
NCT03676959	ZKAB001	Anti-PD-L1	101	Phase 1	Recruiting
NCT03972722	GLS-010 (Zimberelimab)	Anti-PD-1	89	Phase 2	Recruiting
NCT03808857	Geptanolimab (GB226)	Anti-PD-1	80	Phase 2	Recruiting
NCT04623333	TQB2450	Anti-PD-L1	80	Phase 2	Not yet recruiting
NCT03439085	INO-3112 (MEDI0457) + Durvalumab	HPV DNA + Anti-PD-L1	77	Phase 2	Active, not recruiting
NCT04068753	Niraparib + Dostarlimab	Anti-PARP + Anti-PD-1	66	Phase 2	Recruiting
NCT05310383	Tislelizumab + radiotherapy	Anti-PD-1	58	Phase 2	Recruiting
NCT05234905	H101 + Camrelizumab	Recombinant human adenovirus type 5 + Anti-PD-1	55	Phase 2	Not yet recruiting
NCT04341883	Anti-PD-1 + Albumin-Bound Paclitaxel	Anti-PD-1	55	Phase 2	Active, not recruiting
NCT04868708	AK104 (Cadonilimab) ± Bevacizumab + Paclitaxel + Cisplatin/Carboplatin	Anti-PD-1 and CTLA-4 + Anti-VEGF	50	Phase 2	Active, not recruiting
NCT04405349	Atezolizumab + VB10.16	Anti-PD-L1 + HPV16	50	Phase 2	Active, not recruiting
NCT05247619	Tislelizumab + Bevacizumab + Paclitaxel + Cisplatin/Carboplatin	Anti-PD-1 + Anti-VEGF	49	Phase 2	Not yet recruiting
NCT03816553	Camrelizumab (SHR1210) + Apatinib	Anti-PD-1 + Anti-VEGFR-2	49	Phase 2	Active, not recruiting
NCT03912402	BCD-100 (Prolgolimab) + Bevacizumab + Paclitaxel + Cisplatin/Carboplatin	Anti-PD-1 + Anti-VEGF	49	Phase 2	Unknown
NCT03508570	Ipilimumab + Nivolumab	Anti-CTLA-4 + Anti-PD-1	48	Phase 1	Recruiting
NCT03340376	Atezolizumab ± Doxorubicin	Anti-PD-L1	48	Phase 2	Active, not recruiting
NCT05444374	Serplulimab + Bevacizumab + Cisplatin + Paclitaxel	Anti-PD-1 + Anti-VEGF	48	Phase 2	Not yet recruiting
NCT01693783	Ipilimumab	Anti-CTLA-4	42	Phase 2	Active, not recruiting
NCT04380805	AK104 (Cadonilimab)	Anti-PD-1 and CTLA-4	40	Phase 2	Active, not recruiting
NCT04884906	Camrelizumab + Radiotherapy + Albumin Paclitaxel + Cisplatin	Anti-PD-1	40	Phase 2	Recruiting
NCT05475171	MGD019	Anti-PD-1 and CTLA-4	40	Phase 2	Not yet recruiting
NCT04230954	Cabozantinib (XL 184) + Pembrolizumab	Anti-VEGFR + Anti-PD-1	39	Phase 2	Active, not recruiting
NCT04865887	Pembrolizumab + Lenvatinib	Anti-PD-1 + Anti-VEGF	35	Phase 2	Recruiting
NCT04188860	Camrelizumab + Albumin-bound paclitaxel	Anti-PD-1	34	Phase 2	Completed
NCT05179317	QL1706 + Paclitaxel + Cisplatin/Carboplatin	Anti-PD-1 and CTLA-4	30	Phase 2	Not yet recruiting
NCT04641728	Pembrolizumab + Olaparib	Anti-PD-1 + Anti-PARP	28	Phase 2	Recruiting
NCT02257528	Nivolumab	Anti-PD-1	26	Phase 2	Active, not recruiting
NCT03614949	SBRT + Atezolizumab	Anti-PD-L1	26	Phase 2	Recruiting
NCT04551950	M7824 (Bintrafusp alfa) + Cisplatin/Carboplatin + Paclitaxel + Bevacizumab	Anti-TGFβ and PD-L1 + Anti-VEGF	25	Phase 1	Active, not recruiting
NCT03826589	Avelumab + Axitinib	Anti-PD-L1 + Anti-VEGFR	23	Phase 2	Recruiting
NCT04150575	HLX10 (Serplulimab) + Albumin-Bound Paclitaxel	Anti-PD-1	21	Phase 2	Active, not recruiting
NCT03452332	Durvalumab + SABR + Tremelimumab	Anti-PD-L1 + Anti-CTLA-4	20	Phase 1	Active, not recruiting
NCT05310331	Donafenib + Paclitaxel + Platinum ± PD-1 antibody	Anti-VEGFR + Anti-PD-1	20	Phase 2	Recruiting
NCT04635956	Platinum + Etoposide + Bevacizumab + Camrelizumab	Anti-PD-1 + Anti-VEGF	20	Phase 2	Recruiting
NCT05063916	AK104 (Cadonilimab)	Anti-PD-1 and CTLA-4	18	Phase 2	Recruiting
NCT05393440	BS-006 (Recombinant oncolytic type II herpes simplex virus)	Anti-CD3 and PD-L1	18	Phase 1	Not yet recruiting
NCT02921269	Atezolizumab + Bevacizumab	Anti-PD-L1 + Anti-VEGF	11	Phase 2	Complete

Abbreviations: HPV, the human papillomavirus; PD-1, programmed cell death protein 1; PD-L1, programmed cell death ligand-1; CTLA-4, cytotoxic T-lymphocyte-associated protein 4; TIGIT, T cell immunoglobulin and ITIM domains; VEGFR-2, vascular endothelial growth factor receptor 2; VEGF, vascular endothelial growth factor; PARP, Poly (ADP-ribose) Polymerase; SBRT, stereotactic body radiation therapy; SABR, stereotactic radiosurgery; TIL, tumor-infiltrating lymphocyte therapy; HDAC, histone deacetylases; CCR8, chemokine receptor 8; TKI, tyrosine Kinase Inhibitor; ICOS, inducible T-cell co-stimulator; LAG-3, Lymphocyte Activation Gene-3; FGFR, fibroblast growth factor receptor.
