# Supplementary material for: Immune checkpoint blockade for locally advanced or recurrent/metastatic cervical cancer: An update on clinical data
Source: Front Oncol. 2022 Dec 22;12:1045481. doi: 10.3389/fonc.2022.1045481 (PMC9832370; doi:10.3389/fonc.2022.1045481)
Supplement: SUPPLEMENTARY TABLE 3 — A glossary of abbreviations. [file Table_3.pdf]

Abbreviations

**ADC**	antibody-drug conjugate	**MSI**	microsatellite instability
**AEs**	adverse events	**mut/Mb**	mutations per million bases
**ASCO**	American Society of Clinical Oncology	**NCCN**	National Comprehensive Cancer Network
**CC**	cervical cancer	**NK**	natural killer
**CCRT**	concurrent chemoradiotherapy	**NMPA**	China National Medical Products Administration
**CPS**	combined positive score	**ORR**	objective response rate
**CRs**	complete responses	**OS**	overall survival
**CTLA-4**	cytotoxic T-lymphocyte-associated protein 4	**PD-1**	programmed cell death-1
**DCR**	disease control rate	**PD-L1**	programmed cell death ligand-1
**DCs**	dendritic cells	**PFS**	progression-free survival
**DLTs**	dose-limiting toxicities	**PRs**	partial responses
**DMFS**	distance metastasis-free survival	**R/M**	recurrent/metastatic
**DOR**	duration of response	**SCC**	squamous cell carcinoma
**FDA**	U.S. Food and Drug Administration	**SDs**	stable disease
**FIGO**	International Federation of Gynecology and Obstetrics	**SGO**	Society for Gynecologic Oncology
**HPV**	human papillomavirus	**TGF-βRII**	transforming growth factor-β receptor II
**hrHPVs**	high-risk HPV subtypes	**TIGIT**	T cell immunoglobulin and ITIM domains
**ICB**	immune checkpoint blockade	**TILs**	tumor-infiltrating lymphocytes
**ICOS**	inducible T-cell costimulator	**TMB**	tumor mutational burden
**IDO1**	indoleamine 2, 3-dioxygenase 1	**TME**	tumor microenvironment
**irAE**	immune-related adverse events	**TRAEs**	treatment-related adverse events
**LACC**	locally advanced cervical cancer	**TV**	Tisotumab vedotin
**mAb**	monoclonal antibody	**VEGFR-2**	vascular endothelial growth factor receptor-2
